# Something Fishy about Siamese Fighting Fish (*Betta splendens*) Sex: Polygenic Sex Determination or a Newly Emerged Sex-Determining Region?

**DOI:** 10.3390/cells11111764

**Published:** 2022-05-27

**Authors:** Thitipong Panthum, Kitipong Jaisamut, Worapong Singchat, Syed Farhan Ahmad, Lalida Kongkaew, Wongsathit Wongloet, Sahabhop Dokkaew, Ekaphan Kraichak, Narongrit Muangmai, Prateep Duengkae, Kornsorn Srikulnath

**Affiliations:** 1 Animal Genomics and Bioresource Research Unit (AGB Research Unit), Faculty of Science, Kasetsart University, Bangkok 10900, Thailand; thitipong.pa@ku.th (T.P.); kjaisamut10@gmail.com (K.J.); fsciwos@ku.ac.th (W.S.); syedfarhan.a@ku.th (S.F.A.); lalida4536@gmail.com (L.K.); gamewongsathit@hotmail.com (W.W.); ekaphan.k@ku.th (E.K.); ffisnrm@ku.ac.th (N.M.); prateep.du@ku.ac.th (P.D.); 2 Interdisciplinary Graduate Program in Bioscience, Faculty of Science, Kasetsart University, Bangkok 10900, Thailand; 3 Special Research Unit for Wildlife Genomics (SRUWG), Department of Forest Biology, Faculty of Forestry, Kasetsart University, Bangkok 10900, Thailand; 4 The International Undergraduate Program in Bioscience and Technology, Faculty of Science, Kasetsart University, Bangkok 10900, Thailand; 5 Department of Aquaculture, Faculty of Fisheries, Kasetsart University, Bangkok 10900, Thailand; ffisspd@gmail.com; 6 Department of Botany, Kasetsart University, Bangkok 10900, Thailand; 7 Department of Fishery Biology, Faculty of Fisheries, Kasetsart University, Bangkok 10900, Thailand; 8 Laboratory of Animal Cytogenetics and Comparative Genomics (ACCG), Department of Genetics, Faculty of Science, Kasetsart University, Bangkok 10900, Thailand; 9 Center for Advanced Studies in Tropical Natural Resources, National Research University-Kasetsart University, Kasetsart University, (CASTNAR, NRU-KU, Thailand), Bangkok 10900, Thailand; 10 Center of Excellence on Agricultural Biotechnology (AG-BIO/PERDO-CHE), Bangkok 10900, Thailand; 11 Amphibian Research Center, Hiroshima University, Kagamiyama, Higashihiroshima 739-8527, Japan

**Keywords:** betta, *Betta splendens*, DArTseq™, SNP, sex determination

## Abstract

Fishes provide a unique and intriguing model system for studying the genomic origin and evolutionary mechanisms underlying sex determination and high sex-chromosome turnover. In this study, the mode of sex determination was investigated in Siamese fighting fish, a species of commercial importance. Genome-wide SNP analyses were performed on 75 individuals (40 males and 35 females) across commercial populations to determine candidate sex-specific/sex-linked loci. In total, 73 male-specific loci were identified and mapped to a 5.6 kb region on chromosome 9, suggesting a putative male-determining region (pMDR) containing localized *dmrt1* and *znrf3* functional sex developmental genes. Repeat annotations of the pMDR revealed an abundance of transposable elements, particularly Ty3/Gypsy and novel repeats. Remarkably, two out of the 73 male-specific loci were located on chromosomes 7 and 19, implying the existence of polygenic sex determination. Besides male-specific loci, five female-specific loci on chromosome 9 were also observed in certain populations, indicating the possibility of a female-determining region and the polygenic nature of sex determination. An alternative explanation is that male-specific loci derived from other chromosomes or female-specific loci in Siamese fighting fish recently emerged as new sex-determining loci during domestication and repeated hybridization.

## 1. Introduction

Siamese fighting fish (*Betta splendens* Regan, 1910) [1], commonly called Plakat or betta, are popular ornamentals native to Southeast Asia and are well-known for their aggressive behavior, extremely diverse color patterns, and large variation in fin shapes. Siamese fighting fish show sexual dimorphism; males have long ventral and tail fins with vivid and uniform body coloration, whereas females have shorter fins and dull, patterned bodies [2,3]. Further, the males exhibit an aggressive nature [3,4,5,6,7]. Breeding male Siamese fighting fish for local sale and exports for use as ornamentals and in exhibition contests is a profitable business. Siamese fighting fish are ranked among the top two ornamentals in terms of numbers of fish and revenue in Thailand [7,8]. The development of reliable and effective approaches to control the gonadal sex of Siamese fighting fish and produce all-male populations in controlled conditions would be advantageous for breeders. However, a simple, practical approach to generate all-male progenies in Siamese fighting fish has not been achieved thus far [9], with several treatments negatively impacting body length [10]. This highlights the need to take a step back to first clearly understand the sex determination system (SDS) in Siamese fighting fish before moving forward to develop strategies for the mass production of this important aquaculture species.

Inheritance tests, genome scanning, whole-genome sequencing, expression analyses, and gene knockout studies suggest that Siamese fighting fish likely exhibit an XX/XY SDS with homomorphic sex chromosomes [3,11,12,13,14]. The sex locus was found to be located in a very narrow region on chromosome 9 [13,14]. The functional *dmrt1* gene showed male-biased expression before gonad differentiation, with a high level of epigenetic markers in the genome but not for the X chromosome. However, this information was derived from the analysis of only one commercial stock [14]. Teleosts with different geological populations, ecotypes, or varieties are reported to have different SDSs and/or sex determination regions (SDRs) [15,16,17,18]. Studies on more Siamese fighting fish populations are required to confirm this. By contrast, mating and biased sex ratios of offspring postulate that Siamese fighting fish might have both XX/XY or ZZ/ZW SDSs, as well as polygenic sex determination (PSD) influenced by environmental factors such as temperature [14,19]. Previous studies reported sex reversal as either a spontaneous process or a result of artificial induction in Siamese fighting fish [14,20,21,22,23,24,25]. Most teleosts exhibit homomorphic sex chromosomes, and the turnover of sex chromosomes and ability of sex reversal are frequently observed in species lineages [26]. In light of this scenario, we tested our first hypotheses that Siamese fighting fish exhibit an XX/XY sex-chromosome system, which should harbor male-specific loci across several Siamese fighting fish populations. Recent next-generation sequencing technologies have facilitated the discovery of numerous genetic markers in any organism at affordable costs, facilitating investigations of genetic diversity within and between populations [27]. Only a fraction of homologous regions in the genomes of non-model species can be sequenced and genotyped for single-nucleotide polymorphisms (SNPs) to identify the genomic regions of sex-determining loci [16,17,18,28,29,30,31]. In this study, we addressed our first hypothesis using a genome-wide SNP approach to identify novel SNP loci in captive-bred individuals with known phenotypic sex assignment and determine associated regions in Siamese fighting fish. Gene ontology and in silico chromosome mapping with SNP loci were also used to search for homologies with Siamese fighting fish (accession no. GCF_900634795.3) [32] and other vertebrates using comparative genomic analyses.

The vast biodiversity of Siamese fighting fish is now being lost more rapidly than ever before. Invasion by alien species and hybrids introduced into the wild have caused genetic admixture [3,33,34]. Long-term selection in Siamese fighting fish via crossbreeding between different lines or species has impacted their phenotypic variation and commercially valuable attribute of aggressiveness [3,35,36]. Widespread anthropogenic activities of artificial selection have resulted in outbreeding depression for hybrid Siamese fighting fish (*Betta* spp.) between different species/breeds. Genotypic mixtures might appear in the pet market and be distributed around the world. This led us to propose our second hypothesis that the candidate SDRs of Siamese fighting fish observed from different populations might not be conserved as a result of genetic admixture and outbreeding depression. We tested this hypothesis using different commercial and wild Siamese fighting fish populations with the previous candidate marker involved with conserved non-coding elements [14] and our validated sex-specific loci. Our findings provide novel insights into sex chromosomal genomics in teleosts and other vertebrates.

## 2. Materials and Methods

### 2.1. Specimen Collection for Genome-Wide SNP Analysis and DNA Extraction

Siamese fighting fish are indigenous to central Thailand and the lower Mekong. They are mostly known as domesticated ornamentals, originally bred for use in gambling matches [37]. Thais have bred and sold Siamese fighting fish for centuries (primarily the males) for their beauty and fighting abilities. A wide variety of behaviors and morphologies emerged during captive breeding, including variations in aggressiveness, pigmentation, body size and fin shape. Five commercial populations of Siamese fighting fish were collected from local fish farms, totaling 75 samples (40 males and 35 females) of 1-year-old adults averaging 5.7 cm in length. Sex was determined by external morphological characteristics such as fin and body size [38,39,40]. Dorsal fin samples were collected for DNA extraction. Whole-genomic DNA was extracted following the standard salting-out protocol, with slight modifications for different tissues, as described in Supikamolseni et al. (2015) [41]. High-molecular-weight DNA samples were stored at −20 °C until required for the DArTseq™ (Diversity Arrays Technology Pty Ltd., Canberra, ACT, Australia) library construction, as described previously [28]. All experimental procedures were approved (approval no. ACKU63-SCI-007) by the Animal Experiment Committee of Kasetsart University and conducted in accordance with the Regulations on Animal Experiments at Kasetsart University.

### 2.2. DArT Sequencing and Genotyping

The DArTseq methodology followed that of Jaccoud et al. (2001) [42]. The genotyping of multiple loci was performed using DArTseq™ (Diversity Arrays Technology Pty Ltd.) for SNP loci and in silico DArT for PA loci to determine candidate sex-specific/sex-linked loci within males and females. Approximately 100 ng of DNA from each sample was used to develop the DArTseq™ arrays. The DNA samples were subjected to digestion and ligation reactions, as described previously [28,43]. Sequences were processed using proprietary DArTseq™ analytical pipelines [44]. Outputs generated by DArTsoft14 were filtered based on reproducibility values (>3.5), the average count for each sequence (sequencing depth, >5), balance of average counts for each SNP allele (>0.9), and call rate (>0.8; proportion of samples for which the marker was scored as described previously) [28,30].

### 2.3. Genetic Diversity of the Commercial Population Based on SNP Data Analysis

We analyzed the genetic diversities and profiles of the five commercial populations (POP_BSPGIA, POP_BSPW, POP_BSPG, POP_BSPO, and POP_BSPGL) using STRUCTURE 2.3.4 [45]. The total and average allele numbers, F*_IS_*, F*_IT_*, and F*_ST_* using GenAlEx6 [46] were determined, and structural analyses were performed. The number of hypothetical subpopulations (*K*) was estimated using the software through the application of a Bayesian clustering approach to organize genetically similar genotypes into the same subgroups. Seventy-five individual Markov Chain Monte Carlo (MCMC) simulations were conducted for each *K*-value from 1 to 10, with a burn-in length of 50,000, followed by 100,000 iterations as previously described [47]. The log-likelihood of the observed data for each *K*-value was calculated and compared across the range of *K* values. The best *K*-value was estimated based on the membership coefficient (Q) for each individual in each cluster. The Q values indicate the level of relatedness of each accession to various subgroups. The STRUCTURE results were subsequently analyzed by the STRUCTURE HARVESTER application (http://taylor0.biology.ucla.edu/structureHarvester/: accessed on 6 January 2022) [48,49]. GenAlEx was also used to perform PCoA, based on the standardized covariance of genetic distances calculated for the markers under evaluation, using 999 permutations to visualize the clusters of all individuals examined. Genetic structure was also explored with the model-free DAPC using the package ADEGENET 2.0 [50] in R 3.6.2 [51]. DAPC makes no assumptions about population models [50].

### 2.4. Marker Selection and DArT Sequencing Analysis

Sex-specific/sex-linked loci were obtained from the SNP and PA marker analyses of each commercial population. For an XX/XY sex determination system, the SNP and PA loci were sequenced to 70%, 80%, 90%, and 100% using males in separate data sets. The 100% successfully filtered loci were designated as sex-specific loci. The loci categorized in the 70–90% thresholds were considered sex-linked loci [28,30]. An opposite and similar approach was used to target loci based on the ZZ/ZW system. The Hamming distance was calculated to determine the number of combined loci between male and female individuals to identify pairwise differences in SNP and PA loci using the “rdist” function in R version 3.6.2 [51]. The Hamming distance, CATT, and PIC values were used as indices to evaluate the informativeness of the calculated SNP and PA loci as described previously [28,51,52]. The probability of sex-specific/sex-linked loci showing random associations with sex when using a small sample size was estimated using the formula *P*_i_ = 0.5*^n^*, where *P* is the probability for a given locus, *i* is sex-linked, 0.5 is the probability that either a female is homozygous or a male is heterozygous at a given locus, and *n* is the number of individuals sequenced at the locus [28]. The full dataset and the metadata from this publication are available from the Dryad Digital Repository. Dataset, https://doi.org/10.5061/dryad.wh70rxwqd (accessed on 6 January 2022). 

### 2.5. Comparison of Potential Sex-Linked Loci

Significant differences of sex-linked loci (70:30, 80:20, and 90:10) and sex-specific loci (100:0) were analyzed using the Kruskal-Wallis test (*p* < 0.05) for PA and SNP loci; the former used the “stats” package, while the latter used the “PMCMR” package in R [51]. We analyzed the mean heterozygosity and the standard deviation of the loci. Individual candidate loci were plotted using the “glPlot” function in the dartR-R package [51,53]. PCoA using all groups of the sex-linked loci provided a visual representation of the results [28,51].

### 2.6. In Silico Random Commercial Population Testing of Marker Selection and DArT Sequencing Analysis

Marker selection and DArT sequencing analyses were performed using male and female individuals in each commercial population. Monte Carlo methods were applied to randomly test 100,000 times with each 10% of the sampling population providing a probability distribution for SNP and PA loci across different commercial populations (as a reproducibility test) using the “dplyr” and “stringr” functions in R version 3.6.2 [50]. Monte Carlo methods were used to generate models and test particular statistical approaches [54]. The Monte Carlo model we chose had one or more random components, e.g., the model y = x + ε, where ε is some randomly distributed variable [55]. 

#### DNA Marker Validation in Commercial Individuals

A PCR-based approach was applied to verify sex-specific loci in Siamese fighting fish. To develop the DNA markers, candidate sex-specific loci were randomly selected to develop sex-specific PCR-based markers. Ten SNP loci were selected randomly and filtered using the following four criteria: no repeated sequences, one of two alleles in accordance with the base in the Siamese fighting fish reference, SNP-free flanking sequence for PCR-based sequencing primers or SNP present at the 3′-end of the primer for allele-specific primers, and a flanking sequence of ≥50 bp. Ten SNP loci were needed to pass these Monte Carlo filtering methods with the highest score, reflecting the reproducibility of loci and markers across populations. The applicability of the sex-specific PCR assays was further tested and validated using Siamese fighting fish specimens as mentioned above. PCR amplification was performed using 15 μL of 1X ThermoPol buffer containing 1.5 mM MgCl_2_, 0.2 mM dNTPs, 5.0 μM primers, 0.5 U Taq polymerase (Apsalagen Co., Ltd., Bangkok, Thailand), and 25 ng genomic DNA. The PCR protocol was as follows: initial denaturation at 98 °C for 5 min; 35 cycles of 98 °C for 20 s, 58 °C for 45 s, and 68 °C for 2 min; and a final extension at 68 °C for 5 min. The PCR products were visualized by electrophoresis in 1% agarose gel to examine the presence or absence as a consequence of allele-specific DNA markers. For the PCR-based sequencing, the PCR products were purified using FavorPrep GEL/PCR Purification Mini Kit (Favorgen Biotech Corp., Ping-Tung, Taiwan). Nucleotide sequences of the DNA fragments were determined by the DNA sequencing service of First Base Laboratories Sdn Bhd (Seri Kembangan, Selangor, Malaysia). Multiple sequence alignment was used to search the nucleotide sequences of the PCR product with the candidate SNP sequence by log-expectation (MUSCLE) (http://www.ebi.ac.uk/Tools/msa/muscle/: accessed on 6 January 2022) using default parameters to confirm the identity of the amplified DNA fragments. Primer m.CNE.078772F1 (5′-AGTGGCTTGATCCGACACTC-3′), designed based on the conserved sequence of 61 bp and 291 bp belonging to the *drbx1* transposon, was used to amplify conserved noncoding elements. This was used as a positive marker for male Siamese fighting fish across commercial and wild populations, as proposed by Wang et al. (2022) [14]. 

### 2.7. Validation of DNA Markers across Wild Siamese Fighting Fish Individuals

Wild Siamese fighting fish were captured from five different geographical regions in Thailand (Table S1), and whole genomic DNA was extracted as mentioned above. The applicability of the sex-specific PCR assays, including 10 markers and the positive PCR control marker, was also tested and validated with all wild individuals as mentioned above. To address genetic relationships of the wild and commercial specimens, microsatellite genotyping was performed to estimate the genetic diversity and clustering of specimens. Eight microsatellite primer sets developed originally from Siamese fighting fish were sourced from Chailertrit et al., 2014 [56] (Table S2). The 5′-end of the forward primer of each set of primers was labeled with fluorescent dye (6-FAM or HEX; Macrogen Inc., Seoul, Korea). PCR amplification was performed using 15 μL of the 1X ThermoPol buffer containing 1.5 mM MgCl2, 0.2 mM dNTPs, 5.0 μM primers, 0.5 U Taq polymerase (Apsalagen Co., Ltd.), and 25 ng genomic DNA. The PCR protocol was as follows: initial denaturation at 94 °C for 3 min; 35 cycles of 94 °C for 30 s, 52–61 °C for 30 s, and 72 °C for 30 s; and a final extension at 72 °C for 10 min. The PCR products were detected by electrophoresis in 1% agarose gel. To decrease the influence of false alleles, PCR amplification was performed at least thrice for each sample. Fluorescent DNA fragment length analysis was subsequently performed using an ABI 3730XL automatic sequencer (Applied Biosystems, Foster City, CA, USA) at the DNA sequencing service of Macrogen Inc. Allelic size was determined using Peak Scanner version 1.0 software (Applied Biosystems). The genotypic data generated in this study were deposited in the Dryad Digital Repository Dataset, https://doi.org/10.5061/dryad.wh70rxwqd (accessed on 6 January 2022). Micro-Checker version 2.2.3 was used to identify null allelic markers [57]. PIC values were estimated using the Excel Microsatellite Toolkit [58] and calculated for each locus. To compare wild and commercial specimens, Wright’s *F*-statistic for subpopulations within the total population (F*_ST_*) was calculated using Arlequin version 3.5.2.2 [59]. PCoA and DAPC were conducted to determine the genetic structure of the specimens. The model-based clustering method implemented in STRUCTURE version 2.3.3 was also used to determine population structure [45]. The run length was set to 100,000 MCMC replicates after a burn-in period of 100,000 generations, using correlated allelic frequencies under a straight admixture model. The number of clusters (*K*) varied from 1 to 10, with 25 replicates for each value of *K*. The most probable number of bunches was dictated by plotting the log likelihood of the information (ln Pr (*X*|*K*)) [45] over the scope of the tested *K* esteems before choosing the *K* esteem value at which ln Pr (*X*|*K*) settled. The Δ*K* strategy was also applied utilizing Structure Harvester [49].

#### 2.7.1. In Silico Chromosome Mapping

Significant sex-specific/sex-linked loci were aligned to the chromosome-level assembly of Siamese fighting fish (accession: GCF_900634795.3) using NCBI-BLASTn with default parameters [60]. The output mapped file was filtered with the most significant hits (identity, >95%; alignment length, >65 bp) and then parsed using the custom Linux script to generate a karyotype file format for the visualization of a chromosome map. The aligned and karyotype files were formatted and customized as bed files and used to visualize mapping in the CIRCUS tool [61]. An ideogram was also generated from BLASTn alignments of experimentally validated sex-specific/sex-linked loci in Ensembl (https://www.ensembl.org/Multi/Tools/Blast: accessed on 6 January 2022) [62].

#### 2.7.2. Homology Searching

All sex-linked/sex-specific loci that met our criteria were globally BLAST-searched against the National Center for Biotechnology Information (NCBI) database. We then investigated the homologies between the sex-linked/sex-specific loci and the available reference genomes of Siamese fighting fish (accession: GCF_900634795.3) and other teleosts, including Japanese rice fish (medaka) (*Oryzias latipes* Temminck and Schlegel, 1846) [63], (accession no. GCF_002234675.1) [64], zebrafish (*Danio rerio* Hamilton, 1822) [65] (accession no. GCA_000002035.4) [66], and chicken (*Gallus gallus* Linnaeus, 1758) [67], (accession no. AADN00000000.5; International Chicken Genome Sequencing Consortium 2004) [68]. Using the BLASTn program, we used all loci to search the NCBI database (http://blast.ncbi.nlm.nih.gov/Blast.cgi: accessed on 8 January 2022) and RepBase version 19.11 [69] (Genetic Information Research Institute, http://www.girinst.org/repbase/: accessed on 8 January 2022). This is a specialized database with repeated or other significant sequences, and it only reports results with E-values of lower than 0.005. Query coverage requires >55% identity [28].

### 2.8. Functional Annotation and Gene Ontology of Sex-Specific/Sex-Linked Loci

To understand the biological functions of sex-specific/sex-linked loci detected using the genome-wide SNP analysis, we performed functional annotation. BLASTn was performed with all candidate loci against the reference annotation consisting of the gene dataset of Siamese fighting fish [70]. The reference gene dataset was retrieved from the Ensembl database using the Biomart package (Ensembl Genome server. 2022). The BLASTn results were generated as a tabular formatted output file, and only the significant hits (identity >95% and alignment length >65 bp) were retained. All gene sequences from the reference dataset that corresponded to loci with significant hits were extracted and mapped using NCBI-BLASTx against the proteome dataset (including total annotated proteins) of Siamese fighting fish [59]. The proteome dataset was downloaded from UniProtKB/Swiss-Prot [71]. UniProtKB is a collection of functional information on proteins, with accurate, consistent, and rich annotation. Blast2GO v2.0.36 [72] was used to retrieve Siamese fighting fish genes related to GO and reveal biological pathways. The matching transcripts were further processed to detect associated GO terms, describing BPs, MFs, and CCs. UniProtKB, Gramene Proteins Database (GR_protein), and Protein Data Bank (PDB) were used to identify GO categories. The InterProScan (IPS) function in the Blast2GO software was further used to retrieve protein domains and motif information. Blast2GO also produced the enzyme code (EC) numbers for transcripts with an e-value of less than 1 × 10^−5^. Enrichment of ontologies was performed using Fisher’s exact test with *p*-value < 0.05.

#### Determination of Repetitive Elements in Specific Regions

A de novo TE library was generated from specific regions of the chromosome level genome assembly of Siamese fighting fish using EDTA, with the species parameter set to “others” [73]. The inbuilt RepeatModeler (Institute for Systems Biology, Washington, DC, USA) [74] was used to identify the remaining TEs and other repetitive elements that might have been overlooked by the EDTA algorithm (-sensitive 1). TE and repetitive element identification were performed using RepeatMasker (RM; version 1.332) as the compatible version of the standard NCBI blastn program (NCBI/RMBLAST) (version 2.6.0+) search engine. Genomic regions spanning high levels of sex-specific/sex-linked loci were compared against the TE annotation library. TEs and repetitive elements annotated within this specific region were then extracted using bed tools [75]. All sequences of unclassified elements were re-mapped into the whole-genome sequence using D-GENIES [76] to examine their localization. Multiple hits (short reads of ≥100 bp and ≥1 kb genomic reads at an error rate of ∼15%) of unclassified elements with high abundance were pooled as each genomic segment, where they were examined for tandem arrangement within the segment using a dot matrix analysis of the nucleotide sequences with MAFFT version 7. The following parameters were used: (1) a scoring matrix 200PAM/k = 2 and (2) a gap opening penalty of 1.53. The plots and alignments were executed with a threshold score of 39 (E = 8.4 × 10^−11^). (http://mafft.cbrc.jp/alignment/server/: accessed on 8 January 2022). 

### 2.9. Comparative Genomics between Siamese Fighting Fish and Other Vertebrates

Multi-karyotypes of Siamese fighting fish were compared with whole-genome sequences of other vertebrates, namely, medaka fish, zebrafish, western clawed frog, chicken, Anole lizard, and mainland tiger snake, by simulation of the Genomicus and linkage homology portals [77,78]. Genes are represented by small black dots, leading to diagonal lines inside chromosomes, with the order being similar to the reference and line breaks representing rearrangements. They were overlaid with the color of the chromosome of the reference genome where the homologous gene was located, thus pointing to large-scale homologous chromosome segments. 

## 3. Results

### 3.1. Population Structure and Genetic Diversity Analysis of Commercial Siamese Fighting Fish Populations

**Figure 1:**
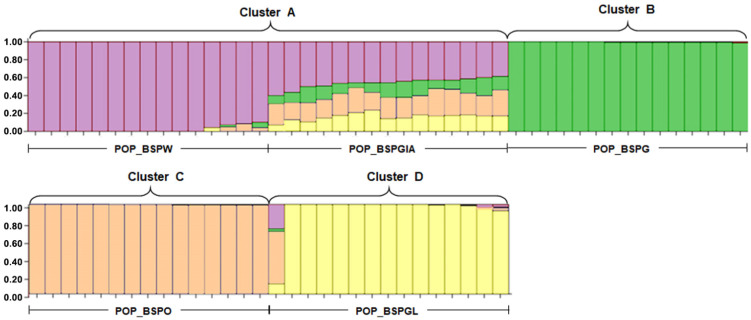
Population structure of 75 samples (40 males and 35 females) of Siamese fighting fish (*Betta splendens* Regan, 1910) [1]. Plot of Evanno’s Δ*K*. Structure bar plots depicting the results of model-based clustering inferred for *K* = 4. Inferred genetic clusters are indicated by different colors. Each vertical bar on the x-axis represents an individual, and the y-axis represents the proportion of membership (posterior probability) in each genetic cluster. Commercial Siamese fighting fish clusters derived from five populations, including cluster A (POP_BSPGIA and POP_BSPW), cluster B (POP_BSPG), cluster C (POP_BSPO), and cluster D (POP_BSPGL).

We sequenced 11,673 SNP loci and 11,836 PA loci (any variability in SNP loci generates presence/absence polymorphism in restriction sites, namely, PA markers) from 75 individuals (40 males and 35 females); 151,257 were alleles observed among all loci, and the mean number of alleles per locus was 0.466 ± 0.002 (Table S3). After 999 permutations, estimates of F*_ST_* showed significant differences between commercial populations (Table S4). AMOVA revealed that the genetic variation was distributed mostly within each population (61% of variation), while only 39% was ascribed to differences among populations. Genetic distances between populations revealed by Nei’s net nucleotide distance (D) indicated a wide distance between POP_BSPG and other commercial populations (Table S5). A principal coordinate analysis (PCoA) revealed that the first, second, and third principal components accounted for 26.83%, 20.76%, and 13.57% of the total variation, respectively, and provided support for four tentatively differentiated commercial Siamese fighting fish clusters derived from five populations, including cluster A (POP_BSPGIA and POP_BSPW), cluster B (POP_BSPG), cluster C (POP_BSPO), and cluster D (POP_BSPGL)) (Figure S1). This was in line with the results of the discriminant analysis of principal components (DAPC) (Figure S1). Bayesian structural analysis revealed the highest posterior probability with one peak (*K* = 4) on the basis of Evanno’s Δ*K*, with all commercial populations grouped into four clusters (Figure 1). This strongly indicated that POP_BSPGIA partially shared the POP_BSPW allelic profile in cluster A. 

### 3.2. Determination of the Sex System and Identification of Sex-Specific/Sex-Linked Loci in Siamese Fighting Fish

**Figure 2:**
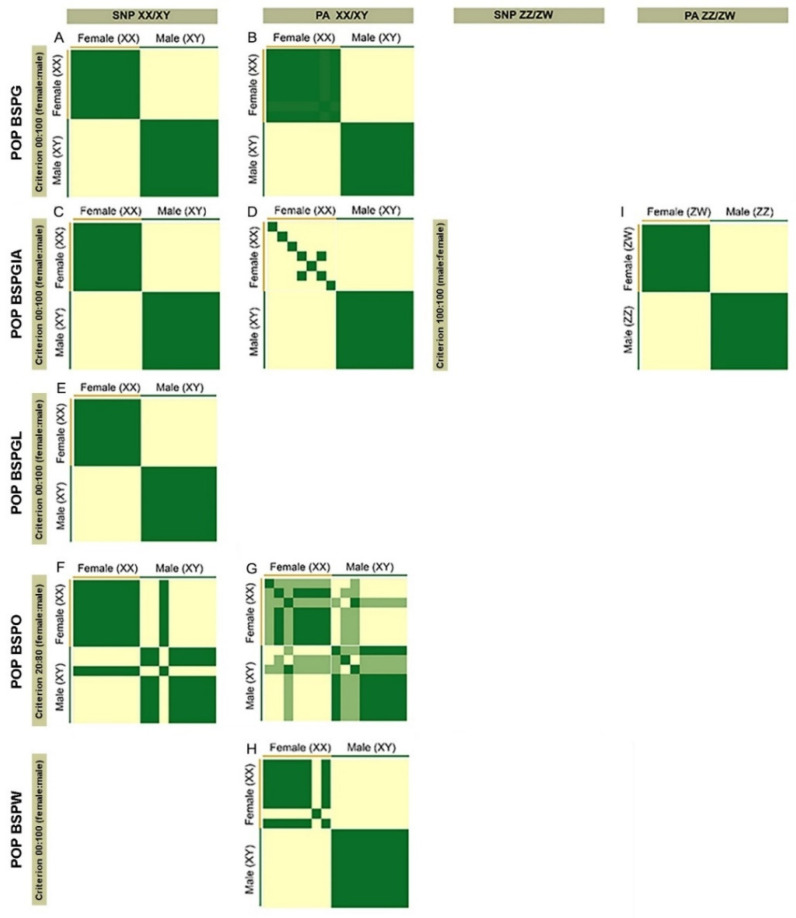
Hamming distances of male and female Siamese fighting fish (*Betta splendens* Regan, 1910) [1] using single-nucleotide polymorphism (SNP) and presence-absence (PA) loci. (**A**) SNP loci filtered with the criterion of 0:100 (females:males) (POP_BSPG), (**B**) PA loci filtered for 0:100 (females:males) (POP_BSPG), (**C**) SNP loci filtered with the criterion of 0:100 (females:males) (POP_BSPGIA), (**D**) PA loci filtered for 0:100 (females:males) (POP_BSPGIA), (**E**) SNP loci filtered with the criterion of 0:100 (females:males) (POP_BSPL), (**F**) SNP loci filtered for 20:80 (females:males) (POP_BSPO), (**G**) PA loci filtered for 20:80 (females:males) (POP_BSPO), and (**H**) PA loci filtered for 0:100 (females:males) (POP_BSPW) for the XX/XY system, (**I**) PA loci filtered for 100:0 (females:males) (POP_BSPGIA for the ZZ/ZW system.

Polymorphic information content (PIC) values ranged from 0.02 to 0.50 (average = 0.26 ± 0.16). For PA, PIC values ranged from 0.00 to 0.50 (average = 0.25 ± 0.16) for SNP. We then compared the number of SNP and PA loci after filtering through criteria with gradual changes. For the XX/XY type, filtering using criteria of 30:70, 20:80, 10:90, and 0:100 (females:males) of five populations presented SNP and PA loci. A Cochran-Armitage trend test (CATT) analysis verified that phenotype was significantly associated with the respective loci, specifically for 73 male-specific loci (Table 1). Hamming distances in males and females are shown in Table 1 and Figure 2. For the ZZ/ZW type, filtering using criteria of 70:30, 80:20, 90:10, and 100:0 (females:males) of five populations presented SNP loci and PA loci. A CATT analysis verified that phenotype was significantly associated with the respective loci containing five female-specific loci (Table 1). Hamming distances in males and females are shown in Table 1 and Figure 2. Kruskal-Wallis tests showed no differences in the percentages of SNP heterozygosity, with filtering criteria in males (H = 2.57, *p* = 0.122) and females (H = 0.55, *p* = 0.321). A PCoA plot revealed that grouping was more similar between the sexes. To investigate the reproducibility of sex-specific loci, 23,509 loci from 75 individuals were repeatedly examined for sex-specific/sex-linked loci with random individual primer sets from different populations using Monte Carlo methods. Results showed 28 sex-specific loci for 3.946% of the sampling time and eight sex-linked loci 0.008% of the sampling time, which was reproducible across all populations (Figure S2).

The range of sample sizes and loci in this study were collected from 75 Siamese fighting fish to minimize the probability of selecting less than one spurious sex-specific/sex-linked marker. The probability of a single locus exhibiting a sex-specific/sex-linked pattern by chance (Pi) was 2.65 × 10^−23^ based on 23,509 loci (including SNP and PA loci). The expected sex linkage was estimated to be 6.22 × 10^−19^. Random sex-linked markers in the commercial Siamese fighting fish population were lower than the expected values.

### 3.3. Validation of Sex-Specific Loci with DNA Markers

**Figure 3:**
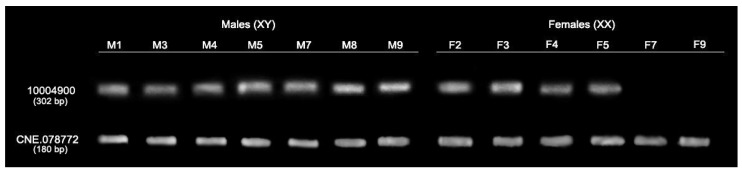
Agarose gel electrophoresis of PCR products in the validation test of sex-linked loci in male and female individuals of Siamese fighting fish (*Betta splendens* Regan, 1910) [1].

A PCR-based method was applied to validate sex-specific loci in Siamese fighting fish. We randomly developed DNA markers from ten loci, and only one locus (locus id: 100004900) was partially validated (Table S6). Two of seven male individuals from the population POP_BSPG were identified with a 302 bp DNA band for the PA100004900 marker (locus id: 100004900), while no specific DNA band was identified in six females (Figure 3). Moreover, when we examined the failure of amplification of sex-specific loci from the primer sets, we found that partial fragments of drbx1 transposable elements (TEs) were amplified in both males and females of all commercial populations as a single 230 bp DNA band. To determine the conservation of sex-linked loci, all sex-linked PCR markers were also applied to wild populations of both male and female Siamese fighting fish.

### 3.4. Genetic Variability across Wild Siamese Fighting Fish Individuals Based on Microsatellite Data

To evaluate the genetic variability in wild and commercial populations, Nei’s net nucleotide distance (D) indicated a wide distance between wild and commercial groups of Siamese fighting fish (Table S7). PCoA revealed that the first, second, and third principal components accounted for 15.65%, 10.26%, and 6.22% of the total variation, respectively, supporting the differentiated wild and commercial groups (Figure S3). This matched with the DAPC and Bayesian structural results (Figures S3 and S4).

### 3.5. Chromosome Localization of Sex-Specific and Sex-Linked Loci

**Figure 4:**
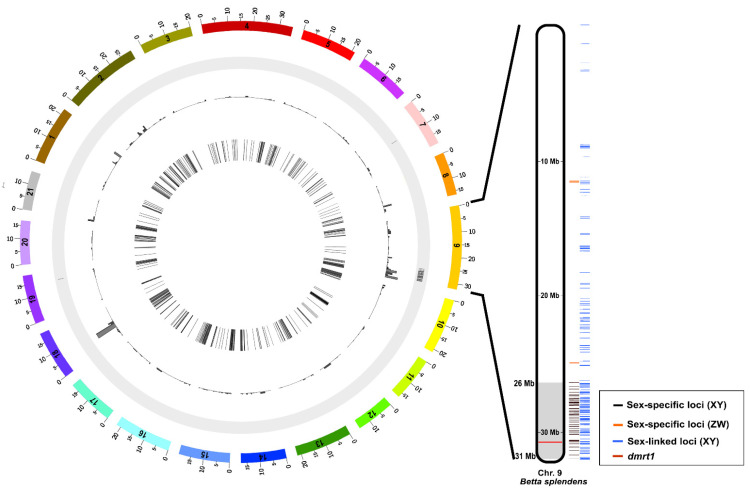
Circos plots showing the distribution of sex-specific and sex-linked loci in Siamese fighting fish (*Betta splendens* Regan, 1910) [1].

In silico chromosome mapping of all sex-specific/linked loci for chromosome-level assembly (accession: GCF_900634795.3) revealed that 71 of 73 male-specific loci were localized to chromosome 9 of Siamese fighting fish (BSP9), while the remaining two loci were mapped on BSP7 and BSP19 (Figure 4, Table S8). Concomitantly, 905 male-linked loci corresponded to BSP9, while 128 male-linked loci were homologous to all chromosomes of Siamese fighting fish. By contrast, five female-specific loci were mapped to BSP9, whereas 81 out of 621 female-linked loci corresponded to BSP9 and 540 to other chromosomes.

### 3.6. Homology of Putative Sex-Specific/Linked Loci

A total of 73 male-specific and 1033 male-linked loci of Siamese fighting fish shared sequence homology with the genomes of Japanese rice fish, zebrafish and chicken. Based on global BLAST analyses using the NCBI databases, 10 of 73 SNP/PA loci on BSP9 were homologous with putative functional genes (Table S9). No loci were included in the sex developmental pathway. No male-linked loci were homologous to any putative functional genes, but some showed partial homology with TEs, while most were Ty3/Gypsy (Figure S5). Notably, six of the seventy three loci on BSP9 demonstrated partial linkage homology of sex chromosomes with other vertebrates. No female-specific loci matched with the putative functional genes and TEs. However, only 4 of the 621 SNP/PA female-linked loci on BSP9 were homologous to Ankyrin repeat and SOCS box protein 15 (ASB15), Refilin-A (RFLNA), shortage in chiasmata 1 (SHOC1), and ATPase phospholipid transporting 8A1 (ATP8A1).

### 3.7. Functional Classification and Enrichment Analysis

**Figure 5:**
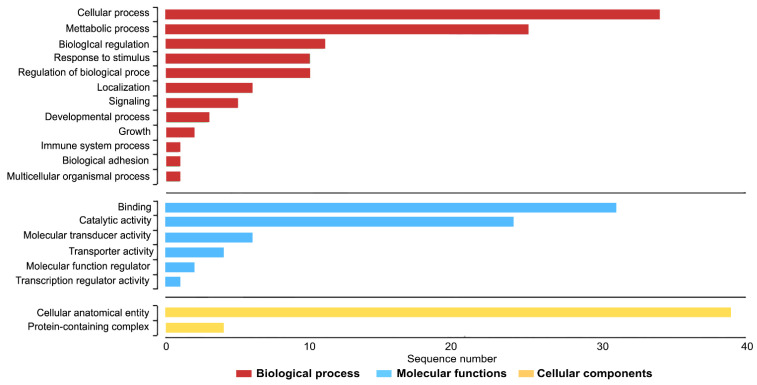
Gene ontology (GO) functional classification of sex-specific loci of Siamese fighting fish (*Betta splendens* Regan, 1910) [1] using Blast2GO. Histograms of the frequency of transcripts annotated to specific GO categories; biological process, molecular functions, and cellular components are represented by red, blue, and yellow bars, respectively.

Gene ontology (GO) enrichment analyses were performed using male-specific loci to classify the putative functions in the comparisons. The GO-enriched categories of biological processes (BPs) were mainly involved in response to cellular process, metabolic process, and biological regulation. GO-enriched categories of molecular functions (MFs) were mainly involved in binding, catalytic activity, and molecular transducer activity. GO-enriched categories of cellular components (CCs) were mainly involved in cellular anatomical entity and the protein-containing complex (Figure 5). By contrast, female-specific loci were classified into the putative functions in the comparisons. The GO-enriched categories of BPs were mainly involved in response to the regulation of cell division, positive regulation of cell division, and signaling. GO-enriched categories of MFs were mainly involved in signaling receptor, carbohydrate derivative binding, and growth factor activity. GO-enriched categories of CCs were mainly involved in cellular anatomical entities and the extracellular region (Figure 5).

### 3.8. Annotation of Repetitive Elements in the Specific Region

Of the 73 male-specific loci, 71 were specifically mapped on BSP9 within 5.6 Mb (locus 26285178–31930103 bp, accession: GCF_900634795.3). This region was screened for TE abundance, and masked areas were recorded by the Extensive de novo TE Annotator (EDTA) [73] and RepeatMasker (RM; version 1.332). Most of these repeats were identified as 48.70% LTR retrotransposons (Ty3/Gypsy and Ty1/Copia), 2.31% Non-LTR retrotransposon (long interspersed nuclear elements (LINEs)), 27.12% DNA transposon (Helitrons, Polintons, and terminal inverted repeats (TIR)), and 21.87% repeat region (unclassified) (Table 2). To extensively investigate the unclassified repetitive elements, all unclassified sequences were characterized and showed nine novel repetitive element types under D-GENIES results. All were predominantly located on BSP9, while a few copy numbers were observed on BSP1 (Figure S6). A dot matrix analysis showed that two of the nine types were tandem-arrayed repetitive sequences (Figure S7). To determine the conservation of the nine types in vertebrates, a homology search of all nine types was performed in medaka fish, zebrafish, and western clawed frog (Xenopus tropicalis Gray, 1864) [79]. Most types were randomly distributed in the genomes of other vertebrates with low copy numbers.

### 3.9. Linkage Homology of Siamese Fighting Fish and Other Vertebrates

Multi-karyotypes of Siamese fighting fish compared with whole-genome sequences of other vertebrates showed that BSP9 was homologous with the sex chromosomes of medaka, zebrafish, and chicken (Figure S8). Notably, BSP9 showed partial homology with chromosome 2 of Anole lizard and mainland tiger snake, while chromosome 2 of squamate reptiles was homologous to chicken Z chromosome.

## 4. Discussion

The primary genetic mechanisms of SDS in Siamese fighting fish remain poorly understood. Recent studies revealed that Siamese fighting fish in one commercial stock have an XX/XY SDS [13], while the dmrt1 gene might be a candidate for sex determination [13,14]. To extend our understanding of the SDS of Siamese fighting fish, we genotyped 75 offspring from five commercial populations at 23,509 loci (without filtering) derived from genome-wide SNPs distributed throughout the genome—on average, one every 18.79 kb. Many false-positive signals might be expected from such specimens because of their diverse genetic backgrounds [80]. We investigated the feasibility of this interpretation. We identified 73 male-specific loci based on genome-wide SNP patterns, indicating part of a putative male SDR. The non-recombination regions of the putative sex chromosome might be cryptic, consistent with species containing homomorphic sex chromosomes [81]. The presence of 1033 male-linked loci suggests a partial recombination region in a small portion of the genome. However, five female-specific loci were also observed in the population POP_BSPGIA, indicating the possibility of female SDR. These results lead us to predict that the SDS of Siamese fighting fish is more complicated than initially believed.

### 4.1. Is Only Chromosome 9 of Siamese Fighting Fish Involved in a SDR?

Sex chromosomes represent a pair of originally homologous chromosomes that diverged by restricting recombination during evolution due to the origin of a sex-determining locus on one member of the pair [82,83]. In this study, 71 out of 73 male-specific loci were identified in 5.6 Mb of the long arm of BSP9 (BSP9q). The 5.6 Mb region is hereafter referred to as a putative male-determining region (pMDR). These 71 loci showed the signature of suppressing recombination and elevated sequence divergence between male and female individuals, suggesting no evidence of balancing selection within the pMDR. This concurs with the phenomenon of homomorphic sex chromosomes, whereas heteromorphic sex chromosomes are sustained by balancing selection if no additional SDS turnover occurs during this evolutionary process [84]. A total of 905 of the 1033 male-linked loci were located on BSP9, with 461 loci (50.939%) identified within the pMDR, thereby suppressing recombination from spreading out from the pMDR to the entire chromosome. This suggests that BSP9 is a sex chromosome at the initial stage of differentiation. Similar cases were also observed in Atlantic salmon (*Salmo salar* Linnaeus, 1758 [85]) [86] and Nile tilapia (*Oreochromis niloticus* Linnaeus, 1758 [87]) chromosomes [88] where sex-specific and sex-linked loci were distributed on the sex chromosomes. The highest repeat area richness of the Siamese fighting fish genome is BSP9, having more than twice the number of repeats than that of the other chromosomes [13]. Repetitive elements can mediate chromosomal rearrangements that further induce sex chromosome differentiation and heterochromatin propagation [3,89,90,91]. Nine novel repetitive element types were observed in the pMDR of BSP9 and in BSP1. By contrast, most types were randomly distributed in the genome of other vertebrates with low copy numbers [92]. This suggests that the nine types might appear and amplify in the pMDR during the sex chromosome differentiation of Siamese fighting fish. Similar findings have been observed in PBI-DdeI satellite DNA distributed in many snake lineages, with high amplification found in the W chromosome of female Siamese cobra (*Naja kaouthia* Lesson, 1831 [93]) [94]. Certain sex-specific loci and a portion of the pMDR demonstrating significant similarity with Ty3/Gypsy were also detected. These are frequently distributed on sex chromosomes in bighead catfish (*Clarias macrocephalus* Günther, 1864) [95] and snakeskin gourami (*Trichopodus pectoralis* Regan, 1910 [1]) [17,28,30,91]. TEs might be involved in sex determination and act as regulatory elements by transcriptional rewiring of the SD regulatory network during the evolution of novel master SD genes [96]. *Dmrt1* was identified in the pMDR of BSP9—like *dmrt1* on sex chromosomes in medaka—while male-biased phenotypic characters and *dmrt1* activity were determined in Siamese fighting fish [17,97]. A 180-bp insertion of uncharacterized TE inserted in intron 4 of X-linked *dmrt1* of Siamese fighting fish was observed with different expression and epigenetic profiles. The same region of X-linked *dmrt1* was applied to develop PCR markers to differentiate male and female individuals [14]. However, in this study, no distinct DNA band patterns were found in either sex from all commercial populations and wild populations, indicating genetic differentiation and different origins. Meanwhile, a 180-bp insertion of TE was reported to have recently emerged only in specific populations during domestication [14]. The mutation underwent the gradual accumulation of sequence variation around *dmrt1* or the insertion site of *drbx1* [14]. This situation is similar to observations in hybrid strains of swordtail fish (*Xiphophorus helleri* Heckel, 1848 [98]), papaya (*Carica papaya* Linnaeus, 1753 [99]), and spotted scat (*Scatophagus argus* Linnaeus, 1766 [100]), where the SDS turned over due to repeated hybridization and selection during domestication [101]. In the present study, one of the ten male-specific loci discovered in Siamese fighting fish was validated in four out of ten individuals of the population POP_BSPG. This probably arose due to population-specific sex-associated loci. The male-specific loci might very recently have undergone sex chromosome differentiation. By contrast, a nonspecific band of the other four populations was detected in female individuals, possibly involved with the stability of the primer-binding site. The remaining nine male-specific markers, detected only in a portion of the specimens, were weakly sex-specific among the adult samples and might be less reliable as sex-linked markers (data not shown). This outcome might be attributed to conserved regions in both sexes of sequences adjacent to sex-specific restriction sites [102]. The failure of the PCR validation step has often been observed after genome-SNP bioinformatics analysis [29,80,103].

The *dmrt1* gene identified in the pMDR and the *znrf3* gene related to the sex developmental pathway seemed reasonable a priori candidate genes for sex determination [104], while 377 other genes were involved with other pathways (Table S10). However, no reports confirm the causative roles of these genes and polymorphisms in sex determination in Siamese fighting fish. The 5.6 Mb region of pMDR would be an important target for future functional investigations. Moreover, 6 out of 461 male-linked loci (id: 100004900, 100020461, 100029210, 100026687, 100001821 and 100017243) were homologous with sex chromosomal linkage in vertebrates [17,28,30]. This result was similar to the comparative homology of sex-specific and linked loci of several teleosts such as bighead catfish, snakeskin gourami, and other amniotes [17,28,30,91]. Using the Ensembl genome browser and Genomicus, comparison of linkage homology of BSP9 with several vertebrates also revealed partial linkage homology with medaka, zebrafish, chicken, Anole lizard, and mainland tiger snake sex chromosomes. These results collectively suggest a super sex chromosome in ancestral amniotes that was shared extensively in teleosts [16,30,82]. Convergent evolution is assumed to be the driving force that causes the divergence of sex chromosomes among phylogenetically distant or closely related taxa [3,82,83]. BSP9 might be enriched with genes contributing to the sex determination cascade or genes with sexually antagonistic effects. At least five functional genes (*dmrt1*, *piwi1*, *piwi2*, *sox5*, and *znrf3*) related to SDS in vertebrates were also identified in BSP9. These genes were homologous to the genes in the sex developmental pathways of other species, despite different SDS such as *dmrt1*. The bird sex chromosome includes the highly conserved *DMRT1*; however, a duplicated copy was identified on the Y sex chromosome of medaka, tongue sole (*Cynoglossus semilaevis* Günther, 1873) [105], and spotted scat [106,107,108]. Interestingly, two out of 73 male-specific loci were located on BSP7 and BSP19 in the POP_BSPGIA and POP_BSPG populations, respectively, suggesting the recent emergence of male-specific loci outside BSP9. We surmised that this could be a signal of the turnover process in different sex chromosomes. The lack of large sex chromosome degeneration may also facilitate further rapid turnover of sex chromosomes. Perhaps, it is simply the combination of multiple and independent “switch” loci or alleles specifying the segregation of sex within a species, known as polygenic sex determination (PSD). PSD can occur by modifying current sex chromosomes to generate a third functional sex chromosome at the same locus, or by modifying autosomal loci in other regions of the genome to generate a novel process for gonad development regulation [16,17,18,109], resulting in the generation of multiple phenotypic or reproductive types within one sex. Such multiple classes may promote fitness benefits for natural selection in the population. PSD systems are evolutionarily unstable because of sex-specific natural selection; they have generally been evaluated as an intermediate step of sex chromosome evolution [110]. This might thus appear in populations where two male-specific loci were observed, i.e., the populations POP_BSPGIA and POP_BSPG. Such male sex-determining genes originate either by duplication followed by neo-functionalization or allelic diversification in Nile tilapia, Northern pike (*Esox Lucius* Linnaeus, 1758 [111]), and medaka (*Oryzias latipes*) [112,113]. Likewise, 128 of 1033 (12.391%) male-linked loci were located in other chromosomes, with eight loci determined on BSP18, while BSP18 contained *foxL2*, *sox3*, *sox8a*, *sox9a*, and *sox9b*. The mammalian *SRY* gene is considered to have evolved from *sox3* as an allelic variant [114,115]. This suggests that the independent recruitment of *sox* is probably required for male determination in relation to PSD. Sex determination pathway mechanisms of different genes might subsequently result from alterations in the effectiveness of interactions with its downstream genes and/or linked *cis*-regulatory elements [116]. This further supports the polygenic nature of Siamese fighting fish sex determination, as well as the occurrence of female-specific loci in the population POP_BSPGIA.

### 4.2. Co-Existence of XY and ZW Sex Determination in Siamese Fighting Fish

SDSs can evolve independently following chromosome structural variation and allelic diversification and promote the rapid turnover of sex chromosomes, leading to SDS differences among closely related species and even among different populations within a species [117]. Here, five female-specific loci and 73 male-specific loci were identified. The female-specific loci were located on BSP9 in the population POP_BSPG but were not found in other populations or in the wild population. This might be explained by frequent recombination within the regions of homomorphic sex chromosomes. Furthermore, female heterozygous sex-linked loci may not always indicate female heterogametic sex determination [118]. Alternatively, the female-specific loci have recently emerged in Siamese fighting fish during domestication. A similar incidence was explained in zebrafish where new loci with young sex determinants evolved [119]. In cichlids, the same chromosome acts as an XY or ZW system in different lineages [19,120,121,122,123,124,125]. TEs may be involved in the rewiring of regulatory networks to adapt to a rapid turnover of SD systems as *cis*-regulatory elements and are also able to directly regulate the expression patterns of key sex-determining genes and drive the turnover of SD systems [96]. A wide TE distribution was observed in the pMDR and their flanking regions on BSP9, implying the relationship between the transitions and turnover of sex chromosomes with TEs [91]. SDS turnover can also be caused by drift [126] or its interaction with sex ratio selection [127]. An alternative explanation is frequent sex-chromosome turnover in teleosts [128,129,130]. Homomorphic sex chromosomes may facilitate frequent sex-chromosome turnover before they degenerate [131,132]. The opposite direction might be that the turnover of SDSs may have occurred in ancestral homomorphic XX/XY sex chromosomes, resulting in the proto-ZZ/ZW group in the population. The family Osphronemidae is one of the largest in the suborder Anabantoidei [133]. Most species have a highly conserved karyotype with a diploid chromosome number (2n) ranging from 42 to 48 [30,134]. Both the XX/XY and ZZ/ZW system remain phylogenetically ancestral in Anabantoidei. In Siamese fighting fish, most male-specific loci and female-specific loci were in the same linkage group, but we can conjecture that this chromosome pair evolved by fusion, as observed in some populations.

In a monofactorial sex determination system, such as the XY chromosome mechanism, the sex ratio is generally 1:1. However, sex ratios in Siamese fighting fish do not always closely approximate to this ideal sex ratio [135]; as in a polyfactorial system, the sex ratio is not necessarily 1:1. Several genes on a number of chromosomes contribute to the sex determination of an individual. Certain individuals may be more strongly male or female than others due to independent segregation and a combination of additive and epistatic effects at these loci [109]. The genetic basis of the SDS in Siamese fighting fish differed among the commercial population in this study, similar to zebrafish, which harbor intraspecific variations for genes influencing sex determination [117]. Sex reversal has also been observed in Siamese fighting fish from different populations [14]. Minor additive effects influenced by environmental factors and discordance of phenotypic sex from the XY system were observed. However, whether these minor genetic loci, in combination with environmental influences, are responsible for sex reversal in Siamese fighting fish remains unclear. Indeed, the explanation of the PSD is most likely, supporting the idea that SDS in Siamese fighting fish contained male-specific loci outside BSP9, together with female-specific loci. High-level dynamism is very important for sexual reproduction and the survival of a species, but sex determination mechanisms are extremely complex and highly variable. To test these hypotheses, potential variations in sex linkage across these loci in different Siamese fighting fish populations or other *Betta* species must be performed to determine how all the known sex-associated regions in BSP9 influence sex and interact in addition to environmental influences.

### 4.3. Genetic Admixture and Introgression of Siamese Fighting Fish Might Complicate Findings

Recent population genetic analyses show that captive populations from farms show remarkable differences from the wild forms [13]. This might reflect the result of breeding plans that collected all dominant traits from different species by backcrossing or hybridization. Different strategies for stock maintenance or genetic drift produced various varieties or interspecific hybrids with different SDSs, while the invasion of hybrids introduced into the wild led to genetic admixture [3]. Multiple alleles from different species might novelly assort into interspecific hybrids, where sets of alleles differ in individuals. Appearances might not be observed in wild individuals of the same species such as in zebrafish, medaka and cichlid fish (*Metriaclima mbenjii*, Stauffer et al., 1997 [136]) [109,137]. This presents a problem for academic research because genetic profiles of Siamese fighting fish from wild or commercial breeds are no longer stable or pure. Perhaps the dynamics of SDS in different populations is nothing more than chance in the admixture within sampled populations, as observed in several local studies in Thailand or international parties, following both official and unofficial research protocols [138]. Unofficial research protocols might also encourage people in the local community to perform different actions such as capture fish from newer locations to receive extra payment. The export of biological materials is heavily regulated and complicated, and the regulations and protocols of the importing and exporting country must be followed. The Nagoya Protocol provides a legal framework for the implementation of the Convention on Biological Diversity and creates transparency for both providers and users of genetic resources. Moreover, it ensures the fair and equitable sharing of benefits arising out of the utilization of genetic resources. In Thailand, researchers must follow practical guidelines issued by the National Research Council of Thailand (NRCT). These guidelines clearly state that before using animals, the user must submit their protocol for approval by the IACUC and the Animals for Scientific Purposes Act B.E. 2258 (A.D. 2015). However, sometimes researchers are caught between prioritizing the ethical implementation of the Nagoya protocol and furthering scientific progress, with some irresponsibly choosing the latter and exploiting the bioresources of developing countries.

Recent developments in massively parallel sequencing technologies and genomic tools have greatly increased the pace of genetic studies on Siamese fighting fish sex determination. A new era is dawning for studying the genetic basis of sex dimorphism, sex determination, and biotechnological manipulation for sex-controlled breeding. Transcriptomic studies and gene targeting and editing approaches have increased the feasibility of functional experiments in fish [139]. This invites new challenges for scientists to develop techniques for the genetic improvement of aquacultural fish species.

## 5. Conclusions

The genomic basis of sex determination is a question of great importance in evolutionary biology. In this study, we carried out genome-wide SNP-based sex genotyping of different commercial Siamese fighting fish populations and identified 71 out of 73 male-specific loci on chromosome 9. We found that Siamese fighting fish sex is mainly determined by an XX/XY system; however, the possibility of the co-existence of female-specific loci cannot be ruled out in some populations, suggesting PSD. Our findings from the annotation of the pMDR showed that, in addition to functional genes, the region is rich in TEs—mainly the Ty3/Gypsy and novel types of repetitive tandem arrays—suggesting that these elements may contribute to the process of sex chromosome differentiation. Finally, our study highlights Siamese fighting fish as emerging model systems to better understand the dynamism of sex chromosomes between domesticated and wild populations due to the impact of differential genetic diversity and admixture.

## Figures and Tables

**Table 1:** DarT analysis of 75 Siamese fighting fish (40 males and 35 females) (XX/XY and ZZ/ZW sex determination type).

		XX/XY Sex Determination Type		ZZ/ZW Sex Determination Type	
		0:100 Female:Male		100:0 Female:Male	
		SNP	PA	SNP	PA
POP_BSPG	Sex-specific loci	2	76	–	–
	Overall mean distance between males and females	1.00 ± 0.000	0.710 ± 0.020	–	–
	Overall mean distance within females	0.502 ± 0.042	0.734 ± 0.043	–	–
	Overall mean distance within males	0.00 ± 0.00	0.520 ± 0.042	–	–
	CATT test	χ2 = 12.451 *p* < 0.001	χ2 = 6.503–14.711 *p* < 0.001	–	–
POP_BSPGIA	Sex-specific loci	1	3	–	3
	Overall mean distance between males and females	1.00 ± 0.00	1.00 ± 0.00	–	1.00 ± 0.00
	Overall mean distance within females	0.00 ± 0.00	0.952 ± 0.048	–	0.00 ± 0.00
	Overall mean distance within males	0.00 ± 0.00	0.00 ± 0.00	–	0.30 ± 0.05
	CATT test	χ2 = 10.452 *p* < 0.001	χ2 = 8.012 *p* < 0.001	–	χ2 = 8.400 *p* < 0.001
POP_BSPGL	Sex-specific loci	1	–	–	–
	Overall mean distance between males and females	1.00 ± 0.00	–	–	–
	Overall mean distance within females	0.00 ± 0.00	–	–	–
	Overall mean distance within males	0.00 ± 0.00	–	–	–
	CATT test	χ2 = 11.258 *p* < 0.001	–	–	–
POP_BSPO	Sex-specific loci	–	–	–	–
	Overall mean distance between males and females	–	–	–	–
	Overall mean distance within females	–	–	–	–
	Overall mean distance within males	–	–	–	–
	CATT test	–	–	–	–
POP_BSPW	Sex-specific loci	–	1	–	–
	Overall mean distance between males and females	–	1.00 ± 0.00	–	–
	Overall mean distance within females	–	0.00 ± 0.00	–	–
	Overall mean distance within males	–	0.00 ± 0.00	–	–
	CATT test	–	χ2 = 13.258 *p* < 0.001	–	–

**Table 2:** Repeat searches for single-nucleotide polymorphisms and restriction fragments of male-linked loci on chromosome 9 in Siamese fighting fish (*Betta splendens* Regan, 1910) [1].

Repeat Class	Fragments	Percentage (%)
LTR retrotransposon	1158	48.70
Ty3/Gypsy	807	33.94
Ty1/Copia	351	14.76
Non-LTR retrotransposon	55	2.31
Long interspersed nuclear elements (LINEs)	55	2.31
DNA transposon	645	27.12
Helitron	512	21.53
Polinton	24	17.09
Terminal inverted repeat (TIR)	109	13.57
Repeat region (unclassified)	520	21.87

## Data Availability

The full dataset and metadata from this publication are available from the Dryad Digital Repository. Dataset, https://doi.org/10.5061/dryad.wh70rxwqd (accessed on 6 January 2022).

## Supplementary Materials

The following supporting information can be downloaded at: https://www.mdpi.com/article/10.3390/cells11111764/s1, Figure S1: Principal component analysis of Siamese fighting fish (*Betta splendens* Regan, 1910) from local farms and discriminant analysis of principal component (DAPC) scatter plots of individuals; Figure S2: Monte Carlo randomization in Siamese fighting fish (*Betta splendens* Regan, 1910); Figure S3: Principal component analysis of wild and commercial groups of Siamese fighting fish (*Betta splendens* Regan, 1910) and discriminant analysis of principal component (DAPC) scatter plots of individuals; Figure S4: Population structure of 75 samples (54 wild and 21 commercial individuals) of Siamese fighting fish (*Betta splendens* Regan, 1910). Plot of Evanno’s Δ*K*. Structure bar plots depicting the results of model-based clustering inferred for *K* = 3. Inferred genetic clusters are indicated by different colors. Each vertical bar on the x-axis represents an individual, and the y-axis represents the proportion of membership (posterior probability) in each genetic cluster; Figure S5: Repeat searches for single-nucleotide polymorphisms and restriction fragments of male-linked loci in Siamese fighting fish (*Betta splendens* Regan, 1910); Figure S6: All copy numbers were intensively located on chromosome 9 of Siamese fighting fish (*Betta splendens* Regan, 1910), with a few observed on chromosome 1; Figure S7: Dot matrix analysis showing that two of nine types had tandem-arrayed repetitive sequences; Figure S8: Multi-karyotypes of Siamese fighting fish (*Betta splendens* Regan, 1910) with whole-genome sequences compared to those of other vertebrates; Table S1: Wild individuals of Siamese fighting fish (*Betta splendens* Regan, 1910) captured from four different geographical regions in Thailand; Table S2: Eight microsatellite primer sets developed from Siamese fighting (*Betta splendens* Regan, 1910) fish by Chailertrit et al. (2014); Table S3: Mean number of alleles per locus based on SNP loci and PA loci from 75 Siamese fighting fish (*Betta splendens* Regan, 1910); Table S4: F*_ST_* showing significant differences between commercial populations of Siamese fighting fish (*Betta splendens* Regan, 1910); Table S5: Genetic divergence among (net nucleotide distance) and within (expected heterozygosity) Siamese fighting fish (*Betta splendens* Regan, 1910) populations, and proportion of membership of population samples; Table S6: Primers used for the development of sex-specific markers for the genotyping assay; Table S7: Genetic divergence among (net nucleotide distance) and within (expected heterozygosity) populations of Siamese fighting fish (*Betta splendens* Regan, 1910), and proportion of membership of wild population samples; Table S8: In silico chromosome mapping aligned to chromosome-level assembly of Siamese fighting fish (*Betta splendens* Regan, 1910) (accession: GCF_900634795.3); Table S9: Gene functions and pathways for restriction fragment single-nucleotide polymorphisms (SNPs) and presence/absence (PA) loci of Siamese fighting fish (*Betta splendens* Regan, 1910) from a BLAST search of Siamese fighting fish, Japanese rice fish (*Oryzias latipes* Temminck and Schlegel, 1846), zebrafish (*Danio rerio* Hamilton, 1822), and chicken (*Gallus gallus* Linnaeus, 1758) genomes (0:100, female:male) (XX/XY sex-determination type); Table S10: Gene pathways in the putative male-determining region (pMDR) of chromosome 9.
