# Supplementary material for: Something Fishy about Siamese Fighting Fish (Betta splendens) Sex: Polygenic Sex Determination or a Newly Emerged Sex-Determining Region?
Source: Cells. 2022 May 27;11(11):1764. doi: 10.3390/cells11111764 (PMC9179492; doi:10.3390/cells11111764)
Supplement: Supplementary file 1 [file cells-11-01764-s001.zip › cells-1741779-supplementary.pdf]

cells

MDPI

Table S1. Wild individuals of Siamese fighting fish (*Betta splendens* Regan, 1910) captured from five different geographical regions in Thailand.

Species	Sample	Sex	Locality
Betta splendens	SP1m1	Male	Bangkok
Betta splendens	SP1m2	Male	Bangkok
Betta splendens	SP1m3	Male	Bangkok
Betta splendens	SP1m4	Male	Bangkok
Betta splendens	SP1m5	Male	Bangkok
Betta splendens	SP1m6	Male	Bangkok
Betta splendens	SP1m7	Male	Bangkok
Betta splendens	SP1m8	Male	Bangkok
Betta splendens	SP1m9	Male	Bangkok
Betta splendens	SP1f1	Female	Bangkok
Betta splendens	SP1f2	Female	Bangkok
Betta splendens	SP1f3	Female	Bangkok
Betta splendens	SP1f4	Female	Bangkok
Betta splendens	SP1f5	Female	Bangkok
Betta splendens	SP1f6	Female	Bangkok
Betta splendens	SP1f7	Female	Bangkok
Betta splendens	SP1f8	Female	Bangkok
Betta splendens	SP1f9	Female	Bangkok
Betta splendens	SP1f10	Female	Bangkok
Betta splendens	SP1f11	Female	Bangkok
Betta splendens	SP1f12	Female	Bangkok
Betta splendens	SP5m1	Male	Chumphon
Betta splendens	SP5m2	Male	Chumphon
Betta splendens	SP5m3	Male	Chumphon
Betta splendens	SP5m4	Male	Chumphon
Betta splendens	SP5m5	Male	Chumphon
**Species**	**Sample**	**Sex**	**Locality**
Betta splendens	SP5f1	Female	Chumphon
Betta splendens	SP5f2	Female	Chumphon
Betta splendens	SP5f3	Female	Chumphon
Betta splendens	SP5f4	Female	Chumphon
Betta splendens	SP5f5	Female	Chumphon
Betta splendens	SP6m2	Male	Surat Thani
Betta splendens	SP6m3	Male	Surat Thani
Betta splendens	SP6m5	Male	Surat Thani
Betta splendens	SP6m6	Male	Surat Thani
Betta splendens	SP6m7	Male	Surat Thani
Betta splendens	SP6f1	Female	Surat Thani
Betta splendens	SP6f2	Female	Surat Thani
Betta splendens	SP6f3	Female	Surat Thani
Betta splendens	SP6f5	Female	Surat Thani
Betta splendens	SP6f6	Female	Surat Thani
Betta splendens	SP6f9	Female	Surat Thani
Betta splendens	SP6f10	Female	Surat Thani
Betta splendens	SPm1	Male	Lamphun
Betta splendens	SPm2	Male	Lamphun

Betta splendens	SPm3	Male	Lamphun
Betta splendens	SPm4	Male	Lamphun
Betta splendens	SPm5	Male	Lamphun
Betta splendens	SPm6	Male	Lamphun
Betta splendens	SPm7	Male	Lamphun
Betta splendens	SPm8	Male	Lamphun
Betta splendens	SPm9	Male	Lamphun
**Species**	**Sample**	**Sex**	**Locality**
Betta splendens	SPf1	Male	Lamphun
Betta splendens	SPf2	Male	Lamphun

Table S2. Eight microsatellite primer sets developed from Siamese fighting fish (*Betta splendens* Regan, 1910) sourced from Chailertrit et al. (2014).

Locus	Primer sequence (5′– 3′)	Repeat	Ta (°C)	Accession	Size (bp)
BettaMS4	F: GTTTCATCAGGAGCAGCAGCATAA<br>R: CTGTTTGATGGCCGACTTTT	$(GA)_n$	59	AB777403	259–315
BettaMS5	F: GTTTCGTCACCTTCTGAGCAAACA<br>R: AAATGCGCTGGGTAGACTTG	$(GA)_n$	59	AB777406	198–218
BettaMS8	F: CGTGAGCTGCAAAGAAAACA<br>R: CTGTTTGATGGCCGACTTTT	$(GA)_n$	57	AB777407	223
BettaMS15	F: ACTGTAACCGGGCTGTTCTG<br>R: AACGCACCCAGAAACAAATC	$(GA)_n$	57	AB777411	216–225
BettaMS17	F: AAGCAGGTCTTTCACCTCCA<br>R: TCACCCTGCGTCTAAGTCAA	$(GA)_n$	61	AB777413	194-221
BettaMS23	F: GTTTGAGAGAAATGGGTTCTTCG<br>R: TCACTACGCTGCCAAATCAG	$(CT)_n(CA)_n$	55	AB777415	259–315
BettaMS25	F: GTTTGGGTAAAACCCAACTCTGG<br>R: AACGTCACGTGGAACAGATG	$(GT)_n$	55	AB777416	194–224
BettaMS40	F: CAGTACATTTGACTGATCGCAGA<br>R: CAGGATGCTTCCTTGGGTAA	$(GA)_n$	57	AB777421	136–165

Chailertrit V, Swatdipong A, Peyachoknagul S, Salaenoi J, Srikulnath K. Isolation and characterization of novel microsatellite markers from Siamese fighting fish (*Betta splendens*, Osphronemidae, Anabantoidei) and their transferability to related species, *B. smaragdina* and *B. imbellis*. Genet Mol Res. 2014; 13: 7157–7162. https://doi.org/10.4238/2014.September.5.1. Regan CT. The Asiatic fishes of the family Anabantidae. Proc Zool Soc Lond. 1910; B1909: 767–787.

Table S3. Mean number of alleles per locus based on single-nucleotide polymorphism (SNP) and presence-absence (PA) loci from 75 Siamese fighting fish (*Betta splendens* Regan, 1910).

	N	Na	Ne	I	Ho	He	uHe	F
**Mean**	**3.693**	**0.466**	**0.466**	**0.000**	**0.000**	**0.000**	**0.000**	
**SE**	0.025	0.002	0.002	0.000	0.000	0.000	0.000	0.000

Regan CT. The Asiatic fishes of the family Anabantidae. Proc Zool Soc Lond. 1910; B1909: 767–787.

Table S4. F_{ST} showed significant differences between commercial populations of Siamese fighting fish (*Betta splendens* Regan, 1910).

Pop1	Pop2	Fst	*p*-value
BSP_G	BSP_GIA	0.465	0.001
BSP_G	BSP_GL	0.658	0.001
BSP_GIA	BSP_GL	0.343	0.001
BSP_G	BSP_O	0.726	0.001
BSP_GIA	BSP_O	0.353	0.001
BSP_GL	BSP_O	0.583	0.001
BSP_G	BSP_W	0.625	0.001
BSP_GIA	BSP_W	0.294	0.001
BSP_GL	BSP_W	0.500	0.001
BSP_O	BSP_W	0.522	0.001

Regan CT. The Asiatic fishes of the family Anabantidae. Proc Zool Soc Lond. 1910; B1909: 767–787.

Table S5. Genetic divergence among (net nucleotide distance) and within (expected heterozygosity) Siamese fighting fish (*Betta splendens* Regan, 1910) populations, and the proportion of membership of the population samples.

Clusters	Net nucleotide distance				Expected heterozygosity	Proportion of membership
	Cluster A	Cluster B	Cluster C	Cluster D		
Cluster A	-	0.1980	0.1380	0.1586	0.21	0.29
Cluster B	0.1980	-	0.2426	0.2387	0.08	0.23
Cluster C	0.1380	0.2426	-	0.1904	0.11	0.26
Cluster D	0.1586	0.2387	0.1904	-	0.13	0.22

Regan CT. The Asiatic fishes of the family Anabantidae. Proc Zool Soc Lond. 1910; B1909: 767–787.

Table S6. Primers used for the development of sex-specific markers for the genotyping assay.

Primer	Sequence (5′–3′)	Size (bp)
PA10004900-F	GGTCATCCTCACACTCCT	302
PA10004900-R	CGTTTTATTGTTCCGGCATT	

Table S7. Genetic divergence among (net nucleotide distance) and within (expected heterozygosity) populations of Siamese fighting fish (*Betta splendens* Regan, 1910), and proportion of membership of wild population samples.

Clusters	Net nucleotide distance			Expected heterozygosity	Proportion of membership
	Cluster A	Cluster B	Cluster C		
Cluster A	-	0.1964	0.1607	0.55	0.28
Cluster B	0.1964	-	0.0466	0.81	0.43
Cluster C	0.1607	0.0466	-	0.86	0.29

Regan CT. The Asiatic fishes of the family Anabantidae. Proc Zool Soc Lond. 1910; B1909: 767–787.

Table S8. *In silico* chromosome mapping aligned to chromosome-level assembly of Siamese fighting fish (*Betta splendens* Regan, 1910) (accession: GCF_900634795.3).

Locus id	Chromosome	Region	%Query Coverage (60%)	E-Value (0.005)	Percent Identity (55%)
PA100003792	7	9381270–9381338	100%	4.00E-28	100.00%
PA100004846	9	27847987–27848032	66%	3.00E-11	93.48%
PA100004464	9	27782844–27782902	92%	3.00E-17	90.62%
PA100029208	9	28233484–28233525	60%	9.00E-11	95.24%
PA100004900	9	30855785–30855834	72%	3.00E-16	98.00%

PA100031354	9	28616136–28616182	68%	2.00E-13	95.74%
PA100004510	9	28543911–28543979	100%	2.00E-26	98.55%
PA100028166	9	27952615–27952678	92%	1.00E-22	96.88%
PA100004858	9	28667903–28667952	72%	3.00E-16	98.00%
PA100004899	9	30159035–30159077	62%	2.00E-12	97.67%
PA100029201	9	29543275–29543327	76%	1.00E-16	96.23%
PA100029211	9	28750932–28750985	78%	3.00E-17	96.30%
PA100004782	9	31334950–31335019	100%	7.00E-25	97.14%
PA100011835	9	28469956–28470024	100%	2.00E-25	97.10%
PA100004843	9	29420491–29420535	68%	1.00E-09	91.11%
PA100027514	9	28423221–28423289	100%	2.00E-25	97.10%
PA100017083	9	29736793–29736861	100%	2.00E-25	97.10%
PA100021008	9	27255876–27255943	98%	7.00E-25	97.06%
PA100012407	9	28875037–28875093	100%	2.00E-13	82.61%
PA100004465	9	27804560–27804625	97%	3.00E-23	97.01%
PA100025925	9	31673853–31673921	100%	2.00E-25	97.10%
PA100004721	9	27067733–27067790	84%	8.00E-18	94.83%
PA100022272	9	27045850–27045901	75%	3.00E-16	96.15%
PA100004670	9	31930035–31930103	100%	8.00E-24	95.65%
PA100004897	9	27316841–27316886	66%	6.00E-13	95.65%
PA100004903	9	31072802–31072845	63%	1.00E-14	100.00%

Locus id	Chromosome	Region	%Query Coverage (60%)	E-Value (0.005)	Percent Identity (55%)
PA100004567	9	27255731–27255795	94%	2.00E-24	98.46%
PA100020889	9	29921025–29921093	100%	5.00E-20	91.30%
PA100004779	9	26334675–26334743	100%	2.00E-26	98.55%
PA100029590	9	29546367–29546416	72%	4.00E-15	96.00%
PA100004412	9	28450542–28450581	89%	2.00E-12	100.00%
PA100021089	9	26285178–26285246	100%	1.00E-22	94.20%
PA100004556	9	30653569–30653635	98%	3.00E-16	90.00%
PA100004680	9	3410145–3410216	100%	1.00E-08	77.78%
PA100030209	9	27689584–27689652	100%	2.00E-25	97.10%
PA100004773	9	29259455–29259523	100%	2.00E-26	98.55%
PA100004645	9	29259455–29259523	98%	1.00E-27	100.00%
PA100004594	9	29740306–29740366	88%	4.00E-21	96.72%
PA100014942	9	28979422–28979490	100%	2.00E-25	97.10%
PA100004619	9	28238728–28238797	100%	3.00E-23	95.71%
PA100004404	9	27876374–27876450	100%	4.00E-22	89.61%
PA100004762	9	30003040–30003107	100%	2.00E-24	97.10%
PA100004801	9	30620699–30620763	100%	6.00E-13	85.51%
PA100004689	9	28266822–28266891	100%	7.00E-25	97.14%
PA100019317	9	30706839–30706907	100%	2.00E-25	97.10%
PA100010245	9	29244547–29244615	100%	2.00E-25	97.10%
PA100004696	9	26797741–26797810	100%	7.00E-25	97.14%
PA100004408	9	28019594–28019662	100%	1.00E-22	94.20%
PA100004563	9	29100891–29100959	100%	1.00E-22	94.20%
PA100004684	9	31129329–31129397	100%	4.00E-28	100.00%
PA100004381	9	27572488–27572556	100%	4.00E-28	100.00%
PA100013910	9	27498305–27498373	100%	2.00E-25	97.10%
PA100020461	9	30600574–30600642	100%	2.00E-25	97.10%
PA100007736	9	31666984–31667052	100%	2.00E-25	97.10%

PA100017244	9	28247164–28247232	100%	2.00E-25	97.10%
PA100024280	9	26618215–26618278	92%	2.00E-25	100.00%
PA100002311	9	29585295–29585363	100%	4.00E-28	100.00%
PA100004745	9	30712428–30712496	100%	4.00E-28	100.00%

Locus id	Chromosome	Region	%Query Coverage (60%)	E-Value (0.005)	Percent Identity (55%)
PA100004685	9	29878539–29878606	98%	7.00E-25	97.06%
PA100004705	9	27696047–27696107	76%	9.00E-11	85.25%
PA100004431	9	27563896–27563965	100%	3.00E-23	95.71%
PA100004615	9	27648339–27648407	100%	4.00E-28	100.00%
PA100004550	9	27231100–27231168	100%	2.00E-25	97.10%
PA100004551	9	30132934–30133001	100%	1.00E-21	94.20%
PA100005701	9	27704093–27704164	100%	2.00E-19	91.67%
PA100015466	9	30427554–30427622	100%	4.00E-28	100.00%
PA100004628	19	15184077–15184147	100%	2.00E-25	97.18%
PA100002103	9	12148504–12148572	100%	4.00E-28	100.00%
SNP100017094	9	28012619–28012551	100%	4.00E-28	100.00%
SNP100017206	9	27467354–27467422	100%	4.00E-28	100.00%
SNP100029210	9	28750985–28750932	78%	5.00E-20	100.00%
SNP100029235	9	29250216–29250263	69%	4.00E-15	97.92%
SNP100007965	9	28686518–28686586	100%	4.00E-28	100.00%

Regan CT. The Asiatic fishes of the family Anabantidae. Proc Zool Soc Lond. 1910; B1909: 767–787.

Table S9. Gene pathways in the putative male-determining region (pMDR) of chromosome 9.

Accession	Start	Stop	Gene symbol
NC_040889.1	26947758	26970809	*aacs*
NC_040889.1	30847795	30861426	*adam9*
NC_040889.1	26390420	26404142	*adamts12*
NC_040889.1	28962819	29033180	*adamts3*
NC_040889.1	30211382	30223171	*adamtsl2*
NC_040889.1	26385900	26390295	*agpat9l*
NC_040889.1	29570659	29619317	*antxr2a*
NC_040889.1	26661903	26665407	*areg*
NC_040889.1	26467158	26537032	*arl15a*
NC_040889.1	29189724	29196923	*ascc2*
NC_040889.1	29474061	29478641	*bmp3*
NC_040889.1	30928254	31034308	*c5*
NC_040889.1	28716487	28734760	*camsap1b*
NC_040889.1	28281143	28293533	*castor1*
NC_040889.1	29388118	29402305	*cds1*
NC_040889.1	26877368	26880870	*cenatac*
NC_040889.1	28844892	28848124	*cfap157*
NC_040889.1	29480720	29530595	*cfap299*
NC_040889.1	31073113	31077836	*ciz1b*
NC_040889.1	27676311	27686895	*ckap2l*
NC_040889.1	29915395	29925731	*cnot6l*
NC_040889.1	27354336	27357287	*coq2*
NC_040889.1	27923912	27934545	*coq5*
NC_040889.1	26553159	26574479	*cspg4ba*
NC_040889.1	28802387	28805356	*ctsla*
NC_040889.1	28755219	28798749	*dapk1*

NC_040889.1	30609989	30628460	*ddr2l*
NC_040889.1	28849529	28864701	*dmrt1*
NC_040889.1	28876321	28880568	*dmrt2a*
NC_040889.1	28868330	28872251	*dmrt3a*
NC_040889.1	27399395	27402291	*emb*
NC_040889.1	31860028	31863675	*endog*
NC_040889.1	26654042	26655286	*epgn*
NC_040889.1	26907697	26913495	*erap1b*
NC_040889.1	30832192	30841733	*ercc6l*
NC_040889.1	29931209	29943567	*fam102aa*
NC_040889.1	28805986	28812813	*fbp2*
NC_040889.1	28324830	28329078	*fbxo21*
NC_040889.1	28309958	28321007	*fbxw8*
NC_040889.1	29532866	29539785	*fgf5*
NC_040889.1	31798841	31808193	*fibcd1a*
NC_040889.1	26439603	26445742	*fsta*
NC_040889.1	26929452	26944939	*gak*
NC_040889.1	26730830	26733022	*gas1a*
NC_040889.1	26376315	26382808	*gck*
NC_040889.1	27334953	27345579	*gldc*
NC_040889.1	29987080	29993754	*gle1*
NC_040889.1	26675986	26677766	*gpx8*
NC_040889.1	30057754	30059936	*gstt1a*
NC_040889.1	26298921	26308788	*hic2*
NC_040889.1	27405370	27407451	*hint2*
NC_040889.1	27704015	27720899	*hs3st1l2*
NC_040889.1	28598582	28602520	*hscb*
NC_040889.1	28018525	28024391	*hspb8*
NC_040889.1	30632390	30633453	*ier3*
NC_040889.1	27583895	27588971	*inpp5e*
NC_040889.1	26696158	26699205	*isca1*
NC_040889.1	26426635	26436919	*itga2.2*
NC_040889.1	27271678	27285028	*jak2b*
NC_040889.1	28817338	28844280	*kank1a*
NC_040889.1	28671809	28712932	*kcnt1b*
NC_040889.1	27881094	27922642	*kremen1*
NC_040889.1	27539101	27545163	*krt1-c5*
NC_040889.1	28177205	28234486	*ksr2*
NC_040889.1	27548017	27554766	*lhx6b*
NC_040889.1	26593906	26598594	*lipg*
NC_040889.1	29925783	29928056	LOC114861258
NC_040889.1	27452464	27463255	LOC114861276
NC_040889.1	27466983	27472812	LOC114861278
NC_040889.1	27463373	27466849	LOC114861280
NC_040889.1	27473105	27475509	LOC114861281
NC_040889.1	27442440	27447386	LOC114861282
NC_040889.1	26600843	26608843	LOC114861288
NC_040889.1	30666683	30676915	LOC114861410
NC_040889.1	30653013	30656027	LOC114861411
NC_040889.1	30646410	30648567	LOC114861412
NC_040889.1	31385686	31400274	LOC114861426

NC_040889.1	31370489	31385568	LOC114861427
NC_040889.1	31230657	31250824	LOC114861429
NC_040889.1	31260556	31266183	LOC114861430
NC_040889.1	31302095	31319545	LOC114861431
NC_040889.1	31275601	31282969	LOC114861432
NC_040889.1	31291908	31300200	LOC114861434
NC_040889.1	31224149	31230556	LOC114861435
NC_040889.1	31358314	31362765	LOC114861437
NC_040889.1	31251062	31260188	LOC114861439
NC_040889.1	31335656	31339880	LOC114861440
NC_040889.1	31331035	31335905	LOC114861441
NC_040889.1	31362807	31371539	LOC114861442
NC_040889.1	31356086	31358532	LOC114861443
NC_040889.1	31266666	31270865	LOC114861445
NC_040889.1	31440505	31447663	LOC114861446
NC_040889.1	31319463	31324329	LOC114861447
NC_040889.1	31275849	31278060	LOC114861449
NC_040889.1	30804291	30806963	LOC114861464
NC_040889.1	29997913	30022057	LOC114861525
NC_040889.1	31580753	31595422	LOC114861556
NC_040889.1	31565052	31580614	LOC114861557
NC_040889.1	31552732	31557198	LOC114861561
NC_040889.1	31557240	31566102	LOC114861562
NC_040889.1	31550514	31552950	LOC114861563
NC_040889.1	28812996	28815962	LOC114861570
NC_040889.1	31867809	31903593	LOC114861577
NC_040889.1	31851389	31867680	LOC114861579
NC_040889.1	31029319	31033178	LOC114861588
NC_040889.1	30089933	30117348	LOC114861590
NC_040889.1	30064696	30068313	LOC114861593
NC_040889.1	30068271	30087324	LOC114861594
NC_040889.1	30110093	30114055	LOC114861596
NC_040889.1	31178484	31209170	LOC114861609
NC_040889.1	31116143	31147296	LOC114861657
NC_040889.1	30711549	30721701	LOC114861672
NC_040889.1	30752529	30756488	LOC114861675
NC_040889.1	30738075	30740691	LOC114861676
NC_040889.1	27766174	27773016	LOC114861677
NC_040889.1	27755119	27764077	LOC114861679
NC_040889.1	27734522	27736067	LOC114861681
NC_040889.1	27738666	27741123	LOC114861682
NC_040889.1	27750508	27753637	LOC114861684
NC_040889.1	30274748	30285907	LOC114861703
NC_040889.1	30365591	30376401	LOC114861704
NC_040889.1	30433406	30438281	LOC114861705
NC_040889.1	30506133	30509450	LOC114861707
NC_040889.1	30287609	30307701	LOC114861709
NC_040889.1	30540752	30542898	LOC114861711
NC_040889.1	30467359	30469509	LOC114861712
NC_040889.1	30514778	30522656	LOC114861713
NC_040889.1	30299217	30300757	LOC114861714

NC_040889.1	26408005	26414414	LOC114861735
NC_040889.1	29957565	29967750	LOC114861744
NC_040889.1	29974360	29986787	LOC114861745
NC_040889.1	29955863	29958574	LOC114861747
NC_040889.1	28614345	28649712	LOC114861749
NC_040889.1	28654120	28656733	LOC114861752
NC_040889.1	31930099	31996736	LOC114861767
NC_040889.1	27808311	27839898	LOC114861770
NC_040889.1	27845137	27847140	LOC114861771
NC_040889.1	27782524	27805310	LOC114861774
NC_040889.1	27875754	27877818	LOC114861776
NC_040889.1	30146110	30156784	LOC114861778
NC_040889.1	30154641	30156738	LOC114861779
NC_040889.1	30148533	30152970	LOC114861780
NC_040889.1	27610443	27639057	LOC114861860
NC_040889.1	29167246	29175112	LOC114861886
NC_040889.1	29202324	29212302	LOC114861887
NC_040889.1	29212445	29252749	LOC114861890
NC_040889.1	30586549	30595663	LOC114861927
NC_040889.1	30577713	30581238	LOC114861928
NC_040889.1	30566912	30577192	LOC114861929
NC_040889.1	28736492	28754834	LOC114861933
NC_040889.1	28712935	28716341	LOC114861934
NC_040889.1	28661460	28672136	LOC114861935
NC_040889.1	28072820	28076922	LOC114861941
NC_040889.1	31053147	31070931	LOC114861942
NC_040889.1	26699335	26703362	LOC114861996
NC_040889.1	26683430	26685607	LOC114861997
NC_040889.1	26680561	26682824	LOC114861998
NC_040889.1	30880203	30891893	LOC114862033
NC_040889.1	27408355	27416296	LOC114862036
NC_040889.1	27416299	27419104	LOC114862040
NC_040889.1	27420058	27427325	LOC114862042
NC_040889.1	29293056	29296494	LOC114862057
NC_040889.1	29320082	29321426	LOC114862058
NC_040889.1	29025341	29027152	LOC114862132
NC_040889.1	27646623	27671252	LOC114862276
NC_040889.1	27590126	27590997	LOC114862284
NC_040889.1	30680994	30689903	LOC114862288
NC_040889.1	30246152	30255132	LOC114862291
NC_040889.1	30255282	30263473	LOC114862293
NC_040889.1	31752107	31775611	LOC114862295
NC_040889.1	31673486	31723501	LOC114862296
NC_040889.1	31788604	31789180	LOC114862300
NC_040889.1	26334747	26363815	LOC114862306
NC_040889.1	29705414	29727371	LOC114862346
NC_040889.1	29698450	29705424	LOC114862350
NC_040889.1	29637292	29676135	LOC114862353
NC_040889.1	29530520	29532877	LOC114862355
NC_040889.1	29324558	29325591	LOC114862360
NC_040889.1	31171627	31173351	LOC114862374

NC_040889.1	29437884	29440048	LOC114862377
NC_040889.1	31456054	31460270	LOC114862421
NC_040889.1	31495478	31513158	LOC114862434
NC_040889.1	31513078	31515114	LOC114862435
NC_040889.1	27696880	27700064	LOC114862463
NC_040889.1	27700591	27703299	LOC114862464
NC_040889.1	27371894	27378521	LOC114862479
NC_040889.1	30197264	30199334	LOC114862486
NC_040889.1	30205252	30211335	LOC114862487
NC_040889.1	27357656	27367042	LOC114862578
NC_040889.1	29766975	29787813	LOC114862579
NC_040889.1	29758944	29765372	LOC114862580
NC_040889.1	29789030	29792817	LOC114862581
NC_040889.1	29728681	29732473	LOC114862582
NC_040889.1	29793209	29907127	LOC114862583
NC_040889.1	26834998	26855055	LOC114862584
NC_040889.1	31630917	31635518	LOC114862627
NC_040889.1	26316289	26317844	LOC114862654
NC_040889.1	27526244	27538207	LOC114862686
NC_040889.1	27505859	27514912	LOC114862689
NC_040889.1	27484897	27496993	LOC114862691
NC_040889.1	27512410	27519323	LOC114862692
NC_040889.1	27497121	27504795	LOC114862693
NC_040889.1	27508882	27516719	LOC114862696
NC_040889.1	30177071	30179990	LOC114862698
NC_040889.1	30180785	30182588	LOC114862699
NC_040889.1	31470113	31478400	LOC114862701
NC_040889.1	31460595	31463081	LOC114862702
NC_040889.1	28369464	28382487	LOC114862722
NC_040889.1	26318239	26320371	LOC114862736
NC_040889.1	26326720	26329139	LOC114862737
NC_040889.1	30234930	30240463	LOC114862743
NC_040889.1	31822691	31845250	LOC114862755
NC_040889.1	26671707	26679047	LOC114862814
NC_040889.1	31485493	31493601	LOC114862846
NC_040889.1	31529546	31533786	LOC114862850
NC_040889.1	31525589	31529795	LOC114862851
NC_040889.1	30902135	30912423	LOC114862886
NC_040889.1	27568828	27576319	LOC114862909
NC_040889.1	26667864	26671346	LOC114862951
NC_040889.1	26922400	26929017	LOC114862952
NC_040889.1	27842434	27843957	LOC114862966
NC_040889.1	30026153	30029523	LOC114862997
NC_040889.1	29732861	29735162	LOC114862998
NC_040889.1	29735164	29736786	LOC114863000
NC_040889.1	29736789	29738807	LOC114863003
NC_040889.1	29738815	29751477	LOC114863004
NC_040889.1	30565811	30566914	LOC114863015
NC_040889.1	30900295	30902003	LOC114863019
NC_040889.1	31165651	31171560	LOC114863022
NC_040889.1	31219153	31224045	LOC114863026

NC_040889.1	31450008	31455586	LOC114863027
NC_040889.1	31732855	31737576	LOC114863036
NC_040889.1	31160382	31165616	LOC114863049
NC_040889.1	26655758	26659333	LOC114863052
NC_040889.1	27320075	27330820	LOC114863188
NC_040889.1	27299839	27309158	LOC114863189
NC_040889.1	27288537	27294893	LOC114863190
NC_040889.1	27266044	27270337	LOC114863194
NC_040889.1	30600248	30604907	LOC114863196
NC_040889.1	31847400	31848113	LOC114863204
NC_040889.1	27674197	27675640	LOC114863211
NC_040889.1	27994212	28001768	LOC114863233
NC_040889.1	31109301	31111435	LOC114863249
NC_040889.1	26881536	26901989	LOC114863263
NC_040889.1	26918672	26921531	LOC114863264
NC_040889.1	31285660	31285769	LOC114863308
NC_040889.1	31644709	31644898	LOC114863315
NC_040889.1	30072301	30075944	LOC121201622
NC_040889.1	31447858	31449395	LOC121202419
NC_040889.1	28307990	28309554	LOC121202429
NC_040889.1	30117397	30121153	LOC121202435
NC_040889.1	30141744	30144781	LOC121202443
NC_040889.1	27213187	27214143	LOC121202446
NC_040889.1	31730535	31731244	LOC121202458
NC_040889.1	31737578	31738607	LOC121202459
NC_040889.1	30186130	30204247	LOC121202467
NC_040889.1	29686911	29688048	LOC121202469
NC_040889.1	26875954	26876507	LOC121202472
NC_040889.1	26590771	26594049	LOC121202490
NC_040889.1	28428414	28431856	LOC121202495
NC_040889.1	29260468	29291460	LOC121202498
NC_040889.1	31271207	31273265	LOC121202500
NC_040889.1	31432951	31439374	LOC121202501
NC_040889.1	30825273	30825967	LOC121202507
NC_040889.1	27996818	27998016	LOC121202509
NC_040889.1	27490583	27497701	LOC121202513
NC_040889.1	30779079	30783521	*lrrc2*
NC_040889.1	28268066	28275438	*lrrc74b*
NC_040889.1	28602521	28610364	*lrrc8ab*
NC_040889.1	30757222	30759779	*mak16*
NC_040889.1	27391370	27399148	*malt1*
NC_040889.1	29148216	29166104	*mapk1*
NC_040889.1	27352199	27354232	*mboat4*
NC_040889.1	26819282	26828425	*mccc2*
NC_040889.1	31903635	31928197	*med27*
NC_040889.1	27367485	27371572	*mfsd10*
NC_040889.1	30771019	30778853	*mfsd14bb*
NC_040889.1	30029680	30040396	*mmp11a*
NC_040889.1	29908845	29914893	*mrpl1*
NC_040889.1	27403052	27405145	*mrps30*
NC_040889.1	26644440	26653343	*mthfd2l*

NC_040889.1	26383150	26384512	*myl7*
NC_040889.1	30231065	30234106	*mymk*
NC_040889.1	26902139	26918595	*naaladl1*
NC_040889.1	26445944	26458479	*ndufs4*
NC_040889.1	28329386	28347895	*nf2a*
NC_040889.1	28275548	28280654	*nipsnap1*
NC_040889.1	28242420	28267889	*nos1*
NC_040889.1	29043635	29057536	*npffr2a*
NC_040889.1	30263524	30266833	*nudt12*
NC_040889.1	30807367	30813917	*nudt18*
NC_040889.1	30862051	30877428	*ogdha*
NC_040889.1	26363941	26369046	*osmr*
NC_040889.1	30060271	30064620	*p2rx7*
NC_040889.1	26418444	26423252	*paip1*
NC_040889.1	28161738	28163157	*pebp1*
NC_040889.1	31776689	31798345	*phf19*
NC_040889.1	27579065	27583112	*pmpca*
NC_040889.1	27475705	27480005	*poli*
NC_040889.1	31621783	31630895	*pomt1*
NC_040889.1	30893092	30895326	*ppiaa*
NC_040889.1	29108839	29124773	*ppil2*
NC_040889.1	28610513	28612049	*ppil3*
NC_040889.1	31034658	31046129	*prdm12b*
NC_040889.1	30376460	30537606	*prdm6*
NC_040889.1	29546429	29551571	*prdm8b*
NC_040889.1	29461597	29473788	*prkg2*
NC_040889.1	27378506	27386628	*prlrb*
NC_040889.1	28389423	28395963	*prodhb*
NC_040889.1	26870745	26874508	*psat1*
NC_040889.1	29993811	29997071	*ptgesl*
NC_040889.1	27561935	27567978	*ptgs1*
NC_040889.1	30172282	30176686	*ptrh1*
NC_040889.1	31645280	31673357	*rapgef1a*
NC_040889.1	29442356	29458876	*rasgef1ba*
NC_040889.1	28173323	28176313	*rfc5*
NC_040889.1	29196458	29201042	*rnf215*
NC_040889.1	28301646	28307996	*rnft2*
NC_040889.1	30707477	30710220	*rsph14*
NC_040889.1	28441507	28472523	*rtn4r*
NC_040889.1	29099077	29108555	*rufy3*
NC_040889.1	29296452	29314938	*rusc2*
NC_040889.1	26369462	26372396	*sb:cb288*
NC_040889.1	27430082	27442135	*scarb2a*
NC_040889.1	30813957	30814767	*sdhaf1*
NC_040889.1	29969400	29972982	*seta*
NC_040889.1	30126043	30160562	*sh2d3cb*
NC_040889.1	29972841	29974309	si:ch211-51h9.7
NC_040889.1	27773124	27780577	si:dkey-13n15.2
NC_040889.1	27848454	27851755	si:dkey-174n20.1
NC_040889.1	26574900	26578221	si:dkey-193c22.1
NC_040889.1	28016406	28017950	si:dkey-1k23.3

NC_040889.1	26990093	27127030	si:dkey-215k6.1
NC_040889.1	30721925	30740670	si:dkey-220o5.5
NC_040889.1	26320571	26326690	si:dkey-88e18.2
NC_040889.1	26610250	26641723	*slc20a2*
NC_040889.1	29948112	29954084	*slc25a25a*
NC_040889.1	30240557	30245001	*slc2a8*
NC_040889.1	29174628	29187900	*slc39a14*
NC_040889.1	29071173	29095819	*slc4a4a*
NC_040889.1	28919916	28947506	*smarca2*
NC_040889.1	30022262	30026145	*smarcb1a*
NC_040889.1	26539583	26547664	*snx18a*
NC_040889.1	30496211	30505238	*snx2*
NC_040889.1	30763437	30771095	*spinb*
NC_040889.1	28298920	28301786	*spring1*
NC_040889.1	30704911	30707333	*srp19*
NC_040889.1	28024516	28062730	*srrm4*
NC_040889.1	28106578	28113348	*suds3*
NC_040889.1	27851835	27865333	*susd2*
NC_040889.1	27521788	27523944	*tal2*
NC_040889.1	28115190	28161250	*taok3a*
NC_040889.1	26756249	26768711	*tars1*
NC_040889.1	28320853	28324709	*tesca*
NC_040889.1	29402297	29406192	*tmem175*
NC_040889.1	26790928	26817060	*tmem8b*
NC_040889.1	30161082	30164577	*tor2a*
NC_040889.1	31786335	31786405	*trnag-ccc*
NC_040889.1	31786698	31786768	*trnag-gcc*
NC_040889.1	27530138	27530209	*trnag-ucc*
NC_040889.1	27530395	27530466	*trnag-ucc*
NC_040889.1	30914429	30923702	*tti2*
NC_040889.1	26685787	26695775	*tut7*
NC_040889.1	27640016	27646046	*ubac1*
NC_040889.1	26329445	26334078	*utp15*
NC_040889.1	28164119	28169110	*vsig10*
NC_040889.1	29334076	29387110	*wdfy3*
NC_040889.1	28169984	28173367	*wsb2*
NC_040889.1	27986075	27988606	*xbp1*
NC_040889.1	26372588	26376263	*ykt6*
NC_040889.1	29128818	29146694	*ypel1*
NC_040889.1	30677843	30680916	zgc:101664
NC_040889.1	28612462	28614211	zgc:101858
NC_040889.1	27331288	27334751	zgc:114041
NC_040889.1	27479902	27486356	zgc:152968
NC_040889.1	30307803	30365163	*znf608*
NC_040889.1	27927599	27975161	*znrf3*

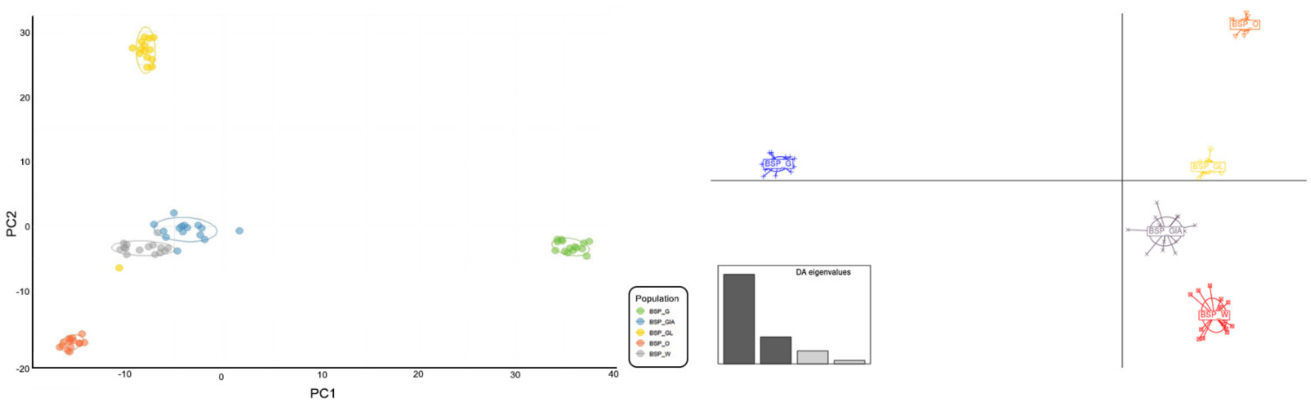

Figure S1. Principal component analysis of Siamese fighting fish (*Betta splendens* Regan, 1910) from local farms and discriminant analysis of principal component (DAPC) scatter plots of individuals.

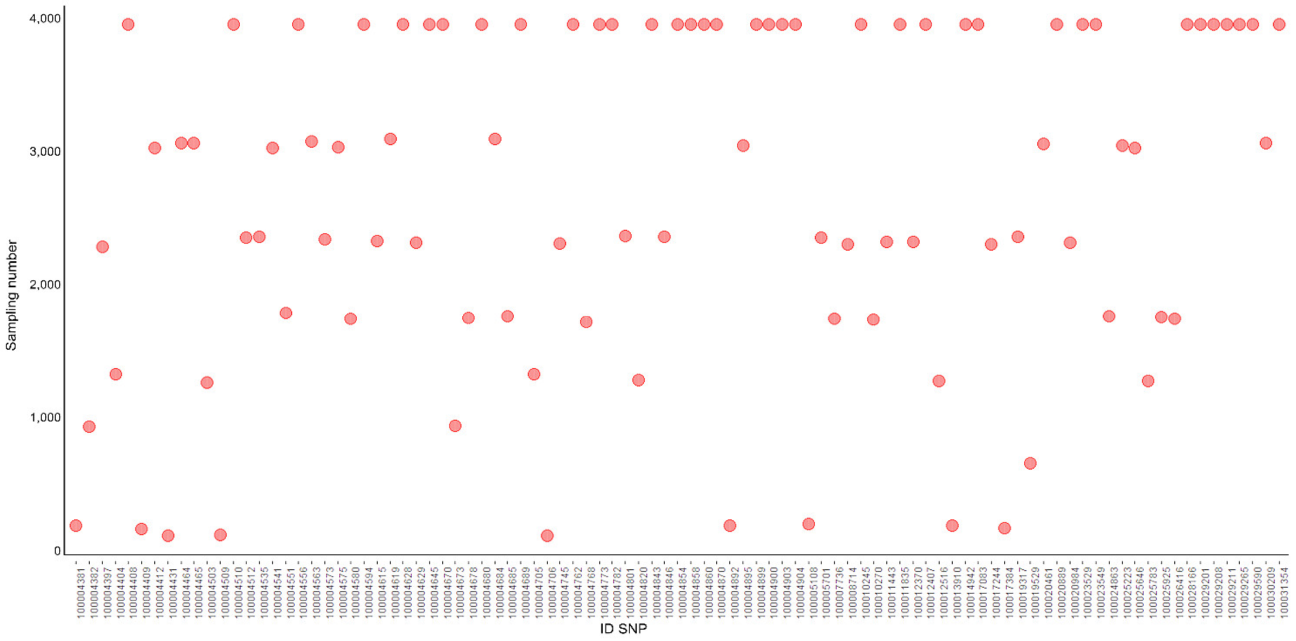

Figure S2. Monte Carlo randomization in Siamese fighting fish (*Betta splendens* Regan, 1910).

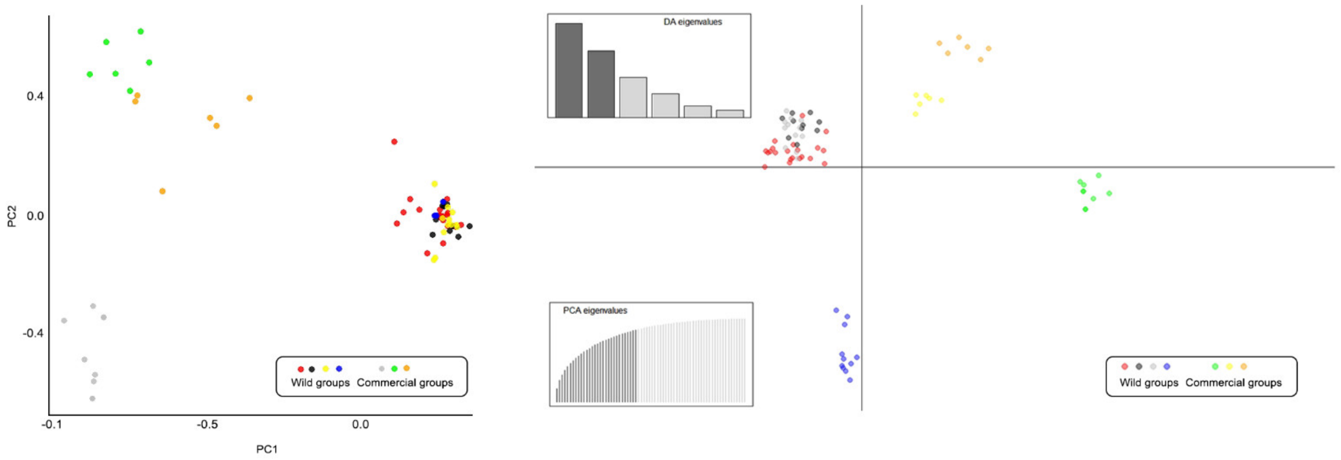

Figure S3. Principal component analysis of wild and commercial groups of Siamese fighting fish (*Betta splendens* Regan, 1910) and discriminant analysis of principal component (DAPC) scatter plots of individuals.

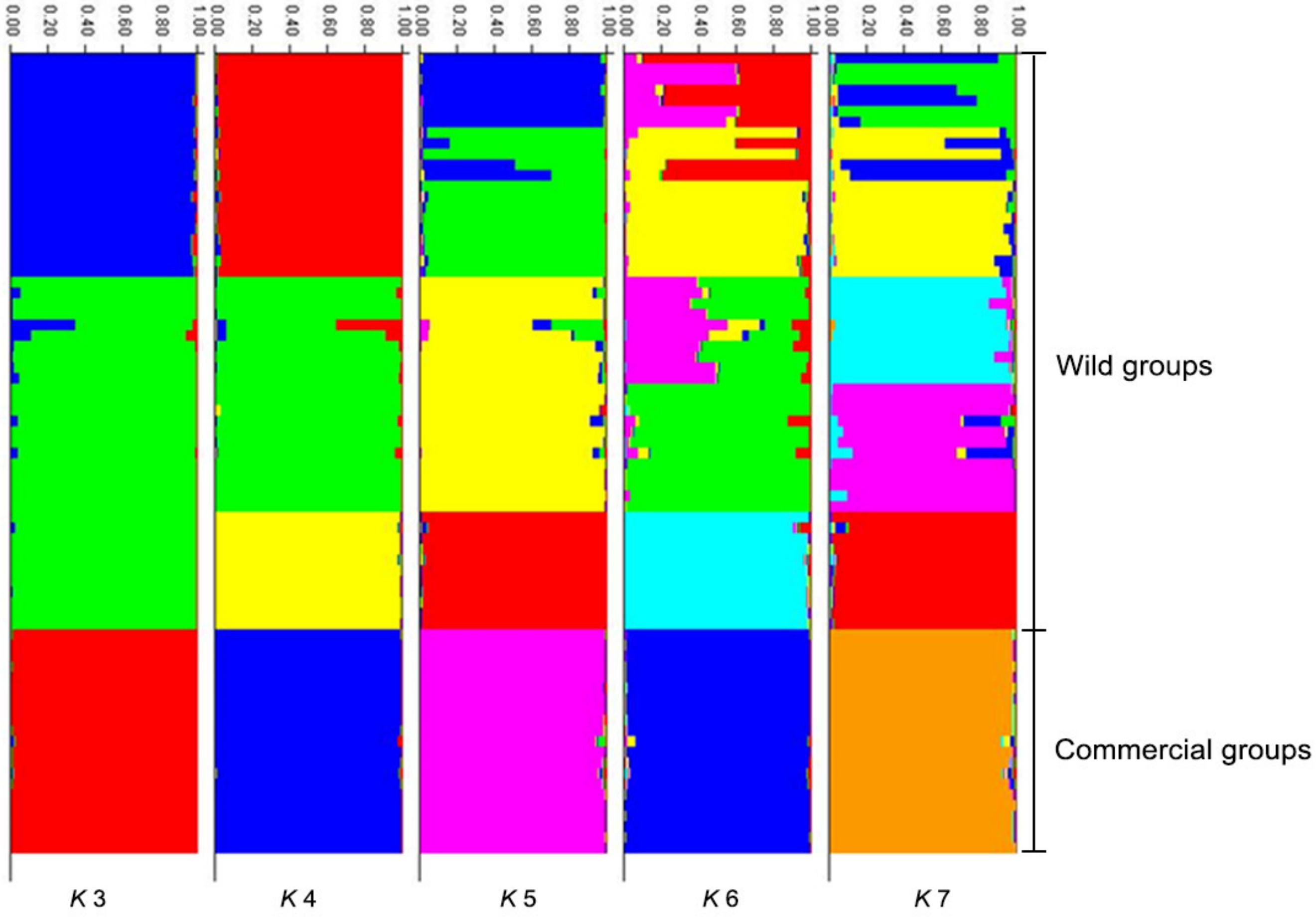

Figure S4. Population structure of 75 samples (54 wild and 21 commercial individuals) of Siamese fighting fish (*Betta splendens* Regan, 1910). Plot of Evanno's Δ*K*. Structure bar plots depicting the results of model-based clustering inferred for *K* = 3. Inferred genetic clusters are indicated by different colors. Each vertical bar on the x-axis represents an individual, and the y-axis represents the proportion of membership (posterior probability) in each genetic cluster,.

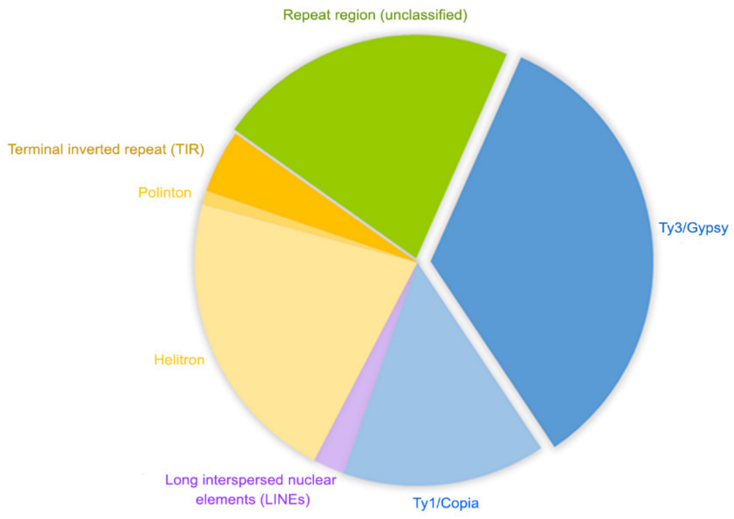

Figure S5. Repeat searches for single-nucleotide polymorphisms and restriction fragments of male-linked loci in Siamese fighting fish (*Betta splendens* Regan, 1910).

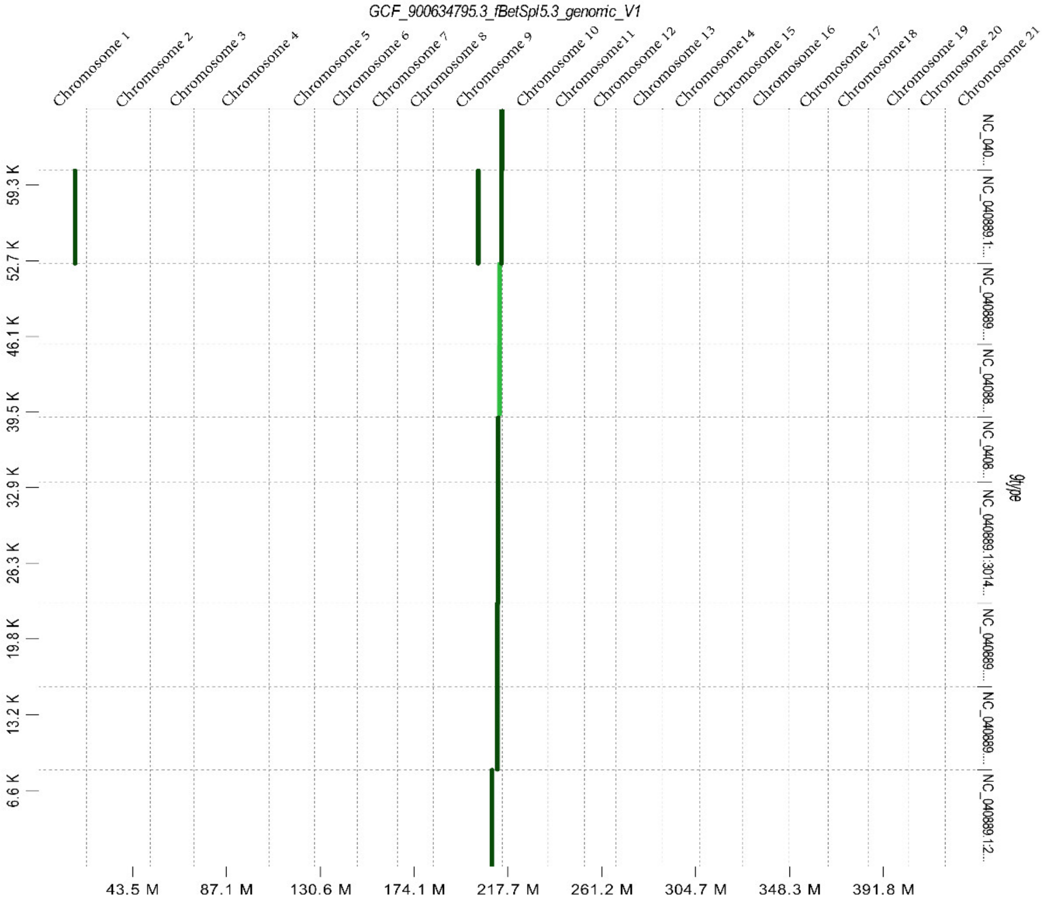

Figure S6. All copy numbers were intensively located on chromosome 9 of Siamese fighting fish (*Betta splendens* Regan, 1910), with a few observed on chromosome 1.

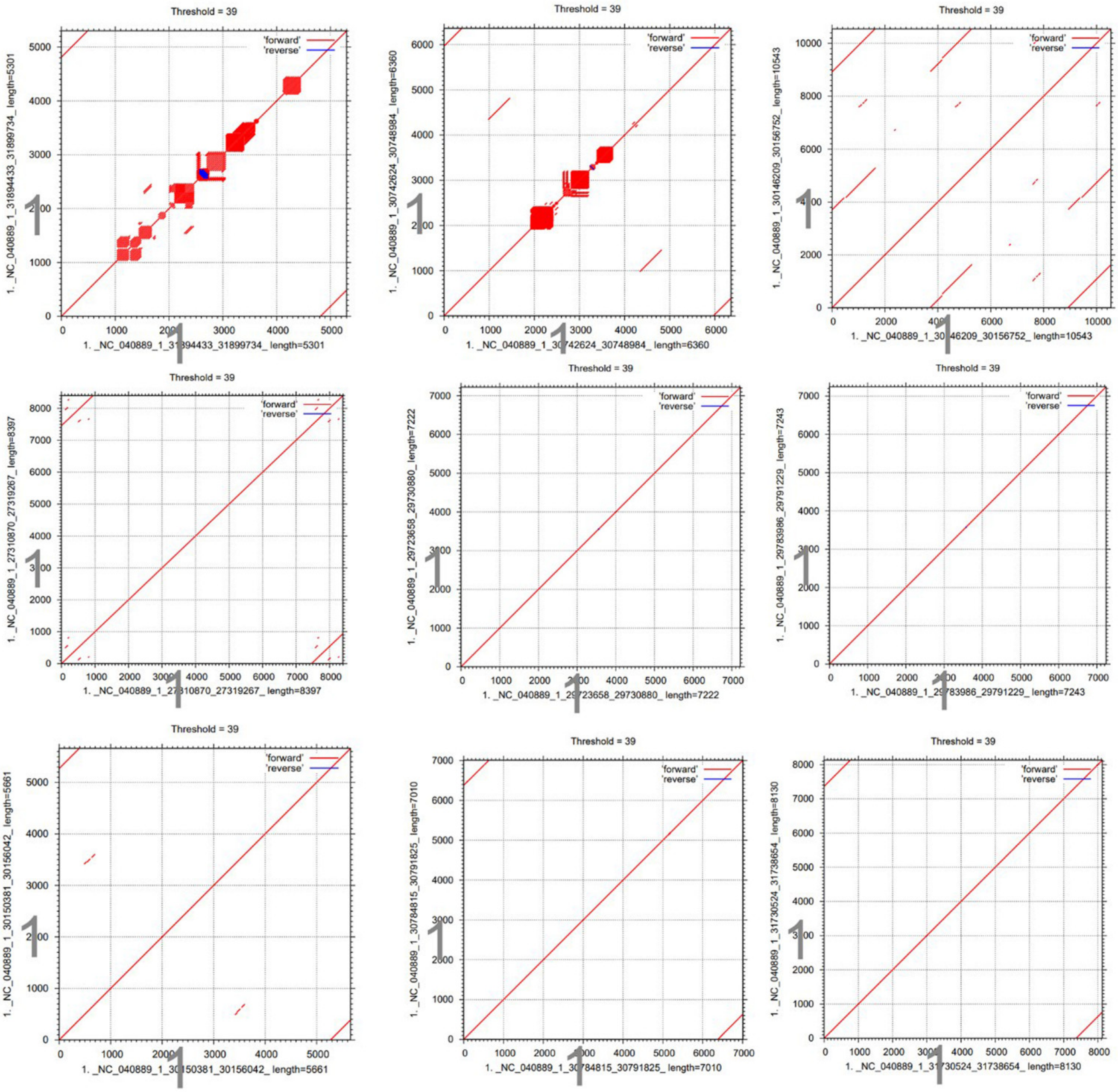

Figure S7. Dot matrix analysis showing that two of nine types had tandem-arrayed repetitive sequences.

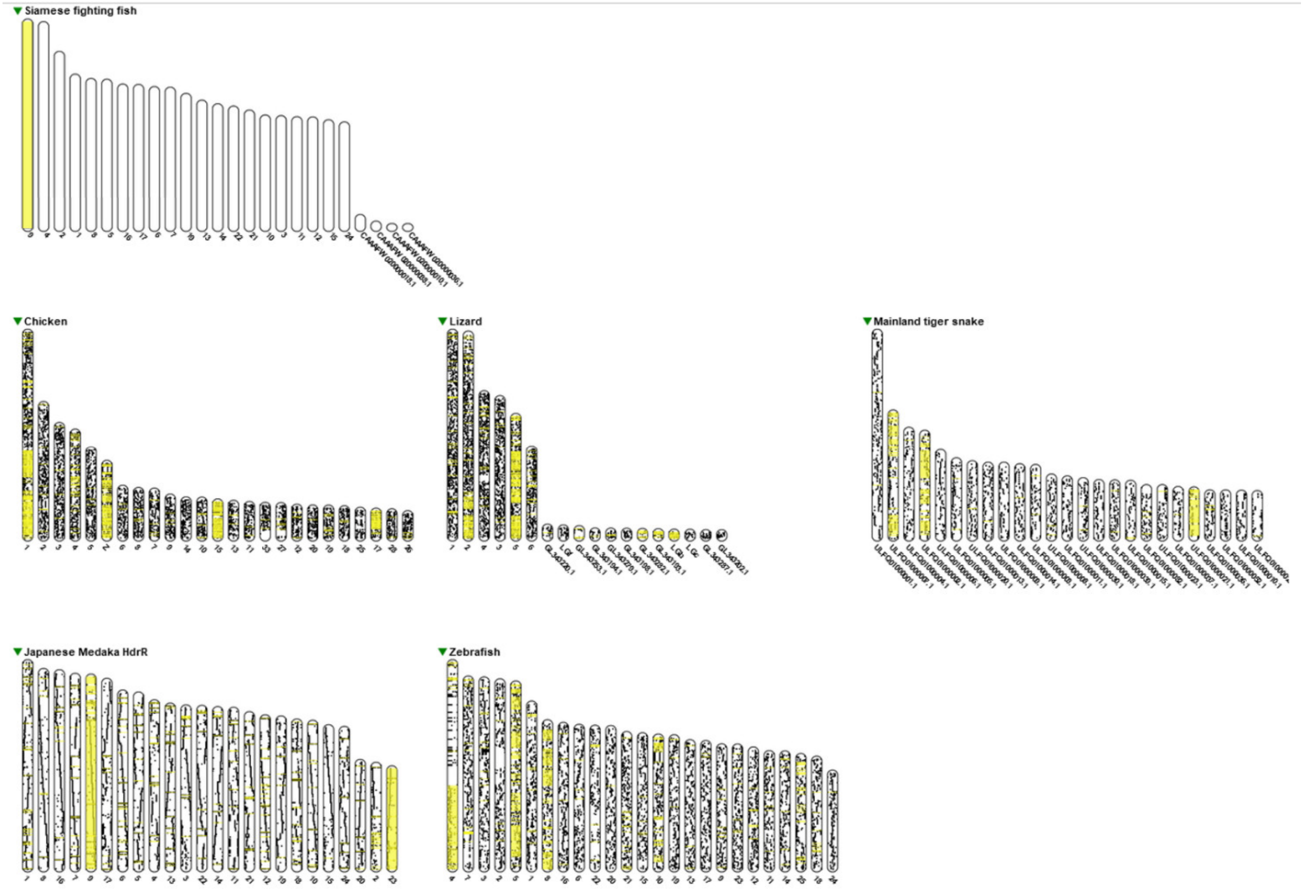

Figure S8. Multi-karyotypes of Siamese fighting fish (*Betta splendens* Regan, 1910) with whole-genome sequences compared to those of other vertebrates,.
